# Phylogenetic analysis of the CDGSH iron-sulfur binding domain reveals its ancient origin

**DOI:** 10.1038/s41598-018-23305-6

**Published:** 2018-03-19

**Authors:** Soham Sengupta, Rachel Nechushtai, Patricia A. Jennings, Jose’ N. Onuchic, Pamela A. Padilla, Rajeev K. Azad, Ron Mittler

**Affiliations:** 1 Department of Biological Sciences, University of North Texas, Denton, TX 76203 USA; 2 The Alexander Silberman Institute of Life Science, Hebrew University of Jerusalem, Edmond J. Safra Campus at Givat Ram, Jerusalem, 91904 Israel; 3 Department of Chemistry & Biochemistry, University of California at San Diego, La Jolla, CA 92093 USA; 4 Center for Theoretical Biological Physics and Department of Physics, 239 Brockman Hall, 6100 Main Street- MS-61, Rice University, Houston, TX 77005 USA; 5 Department of Mathematics, University of North Texas, Denton, TX 76203 USA

## Abstract

The iron-sulfur (2Fe-2S) binding motif CDGSH appears in many important plant and animal proteins that regulate iron and reactive oxygen metabolism. In human it is found in CISD1-3 proteins involved in diabetes, obesity, cancer, aging, cardiovascular disease and neurodegeneration. Despite the important biological role of the CDGSH domain, its origin, evolution and diversification, are largely unknown. Here, we report that: (1) the CDGSH domain appeared early in evolution, perhaps linked to the heavy use of iron-sulfur driven metabolism by early organisms; (2) a CISD3-like protein with two CDGSH domains on the same polypeptide appears to represent the ancient archetype of CDGSH proteins; (3) the origin of the human CISD3 protein is linked to the mitochondrial endosymbiotic event; (4) the CISD1/2 type proteins that contain only one CDGSH domain, but function as homodimers, originated after the divergence of bacteria and archaea/eukaryotes from their common ancestor; and (5) the human CISD1 and CISD2 proteins diverged about 650–720 million years ago, and CISD3 and CISD1/2 share their descent from an ancestral CISD about 1–1.1 billion years ago. Our findings reveal that the CDGSH domain is ancient in its origin and shed light on the complex evolutionary path of modern CDGSH proteins.

## Introduction

The CDGSH domain is part of an iron-sulfur (2Fe-2S) binding motif that appears in several important human proteins e.g., NEET proteins^1–5^. This domain is characterized by the following consensus sequence, [**C**-X-**C**-X2-(S/T)-X3-P-X-**C**-D-G-(S/A/T)-**H**], in which the CDGSH sequence is underlined, and the 3Cys-1His 2Fe-2S coordinating amino acids (aa) are indicated in bold. It was initially annotated as a zinc finger binding domain, but was later shown to bind a 2Fe-2S iron-sulfur cluster^4,6,7^. CDGSH proteins can be classified into Class I CDGSH proteins that contain only one copy of the Fe-S binding domain, and Class II CDGSH proteins that contain two copies of the Fe-S domain^1^. In human, 3 different genes encode CDGSH proteins: CISD1 encodes mitoNEET (mNT), a homodimer that is anchored to the outer mitochondrial membrane (OMM) and is involved in diabetes, obesity, cancer, cardiovascular disease and neurodegeneration^6,8–20^. CISD2 encodes NAF-1, also a membrane-anchored homodimer that is localized to the ER, OMM and the membranes that connect them, and is involved in cancer, neurodegeneration, skeletal muscle maintenance, aging and the regulation of autophagy and apoptosis^21–33^. A NAF-1 dysfunctional variant was also found to be the causative agent of the human monogenic genetic disease Wolfram Syndrome 2 (WFS2) that is associated with juvenile diabetes, hearing deficiencies, neurodegeneration, blindness, and lower life expectancy^34–38^. NAF-1 and mNT were also shown to regulate mitochondrial iron and reactive oxygen species (ROS) metabolism, a function that was proposed to be conserved among plant and animal NEET proteins^4,17,39^. Both mNT and NAF-1 belong to the Class I family of CDGSH proteins^1^. In contrast, CISD3, the third CDGSH human protein, is different from CISD1 and 2 because it is a monomer that contains 2 iron-sulfur (2Fe-2S) clusters, and is hence a Class II CDGSH protein. CISD3 is not membrane anchored, and is localized to the matrix space of the mitochondria^1,2,5^. Very little is known about the function of CISD3 (also known as Miner2), but its expression level was found to be associated with tumorigenesis (http://www.proteinatlas.org). Furthermore, among the 3 human CDGSH proteins, it is the only one to be proposed as an essential protein^40^ (http://tubic.tju.edu.cn/deg/).

The CDGSH domain appears in multiple proteins that belong to bacteria, archaea and many different unicellular and multicellular eukaryotic organisms, often in combination with other important domains such as *Cyt-b5*, *thioredoxin*, *Fer4_*1*9*, *Rieske* and the *Ferritin-like* domain, indicating that it could be involved in various metabolic reactions in different organisms^1^. Perhaps the most important feature of this 3Cys-1His, 2Fe-2S-binding domain, demonstrated for the human CISD1 and CISD2 NEET proteins, is that it is both a relatively stable iron-sulfur binding domain, but at the same time it can participate in different reactions that transfer electrons and/or its entire iron-sulfur cluster to different electron and/or cluster acceptor proteins, respectively^41–51^. This feature may explain why the CDGSH domain is highly conserved from bacteria to human. In addition, it could serve as the basis for the participation of the CDGSH domain in many different important biological functions, as part of essential proteins. A recent study demonstrated, for example, that if the CDGSH domain of NAF-1 is mutated from a 3Cis-1His coordinating structure to a 4Cis coordinating one (a single aa mutation that stabilized the cluster 25-fold over), NAF-1 loses its key function in promoting cellular proliferation in cancer cells^52^. The important function of the CDGSH domain in human disease has also led to different attempts to target this domain with different drugs^9,53,54^.

We recently used the three members of the human NEET protein family (CISD1–3) as guides to conduct a phylogenetic analysis of eukaryotic NEET proteins^1^. Our study suggested that the *Dictyostelium discoideum*’s CDGSH proteins might be the closest to the ancient archetype of eukaryotic NEET proteins. We further suggested that mNT and NAF-1 emerged via gene duplication around the origin of vertebrates^1^. However, the evolutionary timings of these events were not determined. Furthermore, an in depth phylogenetic analysis of the CDGSH domain in bacteria and archaea was not performed.

Here we address the two ends of the CDGSH evolutionary path: The origination of the CDGSH domain in prokaryotes (archaea and bacteria) and eukaryotes; and its divergence times, particularly during the appearance of vertebrates. We show that the CDGSH domain appeared early in evolution, probably linked to the heavy use of Fe-S driven reactions by early organisms^55–58^, and that its early appearance in archaea and bacteria is associated with the ancient 4Fe-4S binding domain *Fer4_19*. We further show that a CISD3-like protein with two CDGSH domains on the same polypeptide (Class II), most likely represents the ancient archetype of CDGSH proteins. We also report that the human Class II (CISD3) protein is more closely related to the bacterial CDGSH protein (of proteobacteria) than to the archaeal Class II CDGSH proteins, and that the human Class I (CISD1/2) protein is more closely related to archaeal than bacterial CDGSH proteins. Using a molecular clock analysis we also show that the separation of the Class I and Class II eukaryotic CDGSH proteins could be traced to the origins of eukaryotic organisms, and that the human mNT and NAF-1 proteins diverged from their common ancestor ~650–720 million years ago (MYA).

## Results

### Occurrence of the CDGSH domain in Archaea

**Figure 1:**
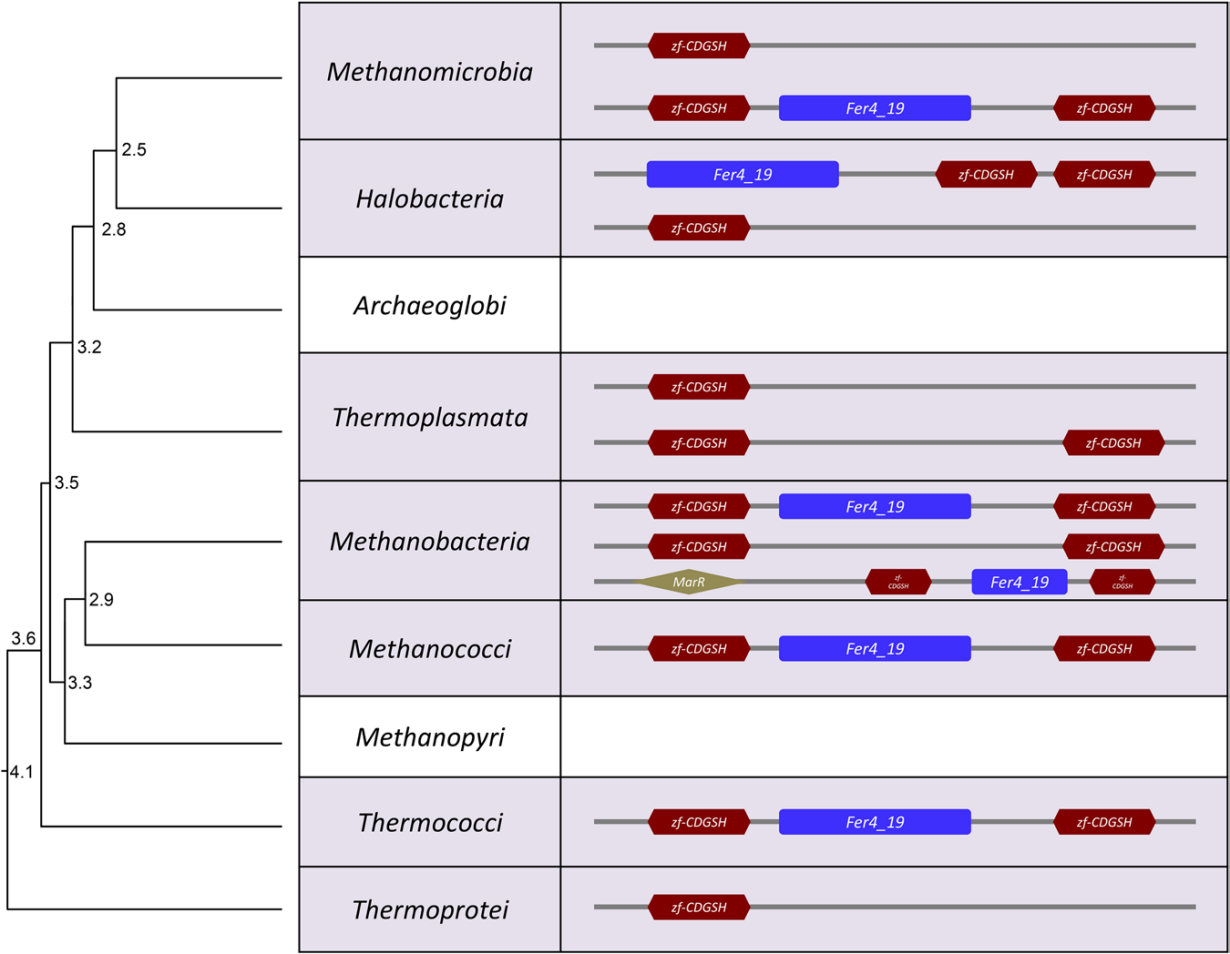
Occurrence and organization of the CDGSH domain in Archaea. A time tree for all archaea classes was obtained from TimeTree.org. The presence or absence of the CDGSH domain within each class was determined using the *Dictyostelium* CISD (XP_647247.1) sequence as the query sequence to perform PSI-BLAST. Archaeal CISD homologs in each class were subjected to PFAM domain analysis to identify the domain organization (see methods). Node ages are represented in billion years ago (BYA).

The CDGSH domain is found in the genomes of extant archaea belonging to different taxa (Fig. 1). Similar to eukaryotic organisms^1^, representatives of both Class I and Class II CDGSH proteins could be found in Archaea (Fig. 1). In addition, in several of the Class II CDGSH proteins of Archaea, the CDGSH domain was found in association with a member of the ancient Fer (*Fer4_19)* 4Fe-4S cluster binding domain^57^. As in eukaryotes^1^, several classes of Archaea lack CDGSH proteins suggesting that some environmental adaptations or metabolic dependencies may not require CDGSH proteins. To conduct a more detailed analysis of CDGSH proteins in archaea, we constructed a phylogenetic tree using PhyML (see Methods) for several different representatives of archaeal CDGSH proteins using the human CISD3 as an outlier to root the tree (Supplementary Fig. S1). As can be seen in Supplementary Fig. S1, the phylogenetic tree of archaeal CDGSH sequences suggests that Class II archaea CDGSH proteins with the *Fer4_19* domain appear to be the most derived (e.g. *Methanococci* and *Methanobacteria*), whereas the Class I CDGSH proteins of *Thermoprotei* appeared to be the among the least derived (the term “derived” refers to branching events in a lineage following divergence from the last common ancestor in a phylogenetic tree). We also retrieved a time tree from TimeTree.org for these proteins (Supplementary Fig. S2). Interestingly, according to the time tree of Archaea (Supplementary Fig. S2), *Thermoprotei*, *Thermococci*, *Methanococci* and *Methanobacteria* appear to have diverged from common ancestors that could be traced back ~3.6–3.9 billion years ago (BYA). This finding could suggest that Class II with the *Fer4_19* and Class I CDGSH domain structure proteins were among the earliest to appear in Archaea. It is also possible that the *Thermoprotei* lineage initially had a Class II with the *Fer4_19* domain protein which was lost in the course of evolution, and only the single domain CDGSH protein was retained. Interestingly, very few examples of Class II CDGSH proteins without a *Fer4_19* domain could be found in archaea. The main forms of archaea CDGSH proteins included therefore the Class I and the Class II CDGSH proteins that contained the *Fer4_19* domain (either in the middle or in the N-terminal).

### Occurrence of the CDGSH domain in bacteria

**Figure 2:**
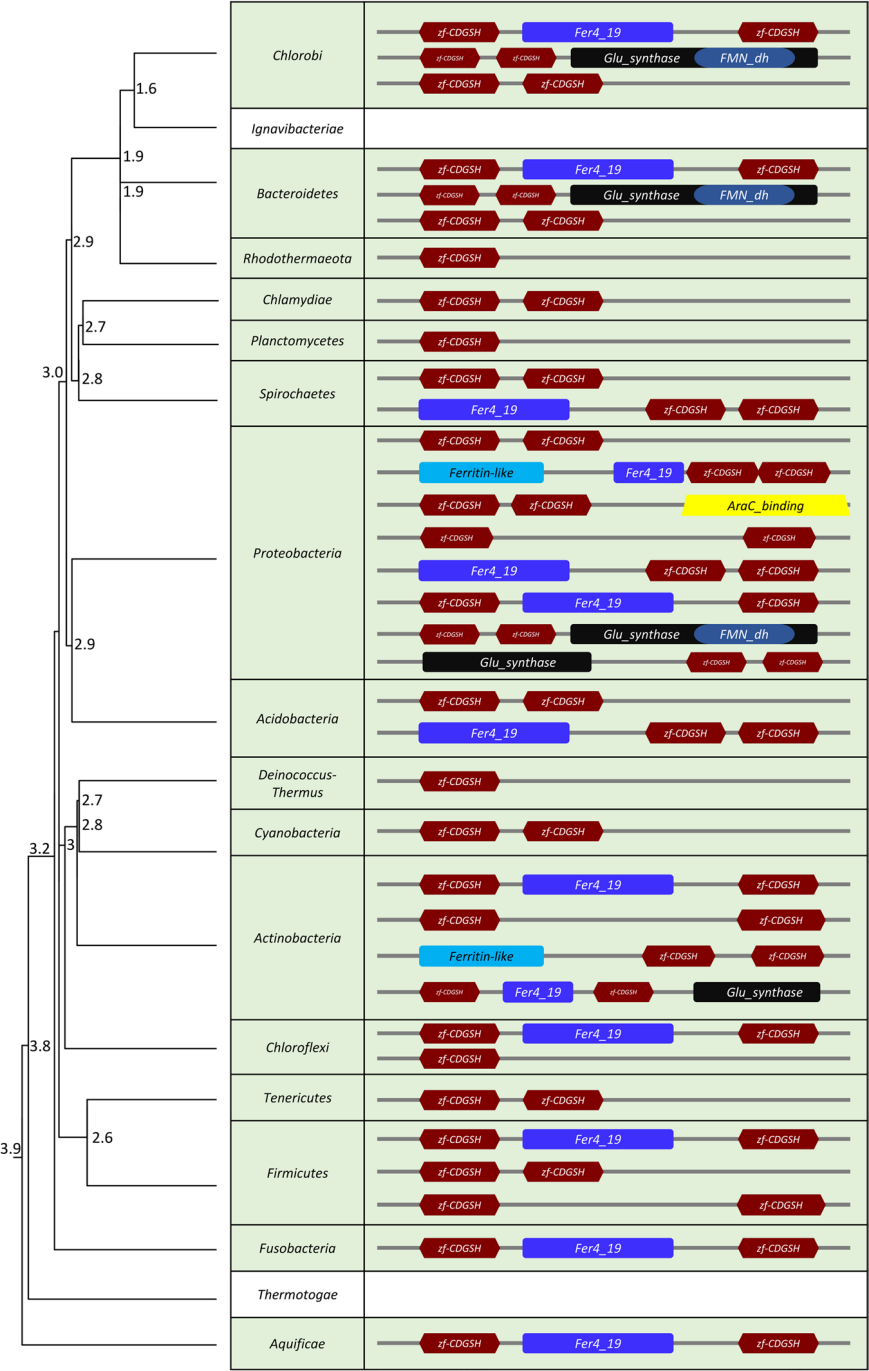
Occurrence and organization of the CDGSH domain in Bacteria. A time tree for all bacteria phylum was obtained from TimeTree.org. The presence or absence of the CDGSH domain and its organization within each phylum was determined using the *Dictyostelium* CISD (XP_647247.1) sequence as the query sequence to perform PSI-BLAST. Bacterial CISD homologs from each phylum were subjected to PFAM domain analysis to identify the domain organization (see methods). Node ages are represented in billion years ago (BYA).

An analysis of CDGSH proteins in different bacterial phyla reveals that the dominant form of CDGSH proteins in bacteria is the Class II type CDGSH protein with two iron-sulfur binding domains present within the same polypeptide (Fig. 2). In contrast, Class I type CDGSH proteins that contain only one CDGSH domain appear in only a few phyla including Rhodothermaeota, Planctomycetes, and the radiation-resistant bacteria *Deinococcus-Thermus*. In many of the bacterial phyla the CDGSH domain appeared in association with other domains such as *Fer4_19*, *Glu_synthase*, *Ferritinlike*, and *Rieske_2*, suggesting that it is associated with many different pathways that are mostly linked to iron and iron-sulfur metabolism (Fig. 2). As with archaea (Fig. 1) and eukaryotes^1^, several phyla of bacteria lack CDGSH proteins suggesting that some metabolic adaptations and/or energy pathways may not require CDGSH proteins. Alternatively, functions similar to those of CDGSH proteins in these bacterial phyla could have been performed by a different class of proteins. To conduct a more detailed analysis of CDGSH proteins in bacteria, we generated a phylogenetic tree of several different representatives of bacterial CDGSH proteins using PhyML with human CISD3 as an outlier to root the tree (Supplementary Fig. S3). The phylogenetic pattern indicated the Class II CDGSH proteins with two CDGSH domains and no other known domains, as in the Proteobacteria *Candidatus Pelagibacter ubique*, are the most derived ones, in contrast to those harboring other domains in addition to the two CDGSH domains, e.g. *Fer4_19* and CDGSH domains protein as in Proteobacteria *Octadecabacter arcticus*, which appears least derived and could potentially be the ancient archetype of bacterial CDGSH protein. The phylogenetic tree also highlights the gain and loss of domains in the evolution of bacterial CDGSH proteins, which even involved the loss of one of the CDGSH domains (Supplementary Fig. S3). Additionally, we observed that the presence of Class II CDGSH domains is restricted to only a few cyanobacteria (by performing PSI-BLAST as mentioned in Methods section), which have grouped largely with proteobacteria. Two cladistic patterns were observed, one group of cyanobacterial CDGSH protein sequences grouping with representatives of proteobacterial CDGSH protein sequences, both with only two CDGSH domains, while another group of cyanobacterial sequences grouped with representatives of proteobacterial sequences, both with an integrated *Glu_syanthase* and *FMN_dh* domain. High bootstrap confidence on these clades suggests that these cyanobacterial CDGSH genes might have been acquired from proteobacteria via horizontal gene transfer. We also retrieved a time tree from TimeTree.org for these proteins (Supplementary Fig. S4). As opposed to archaea (Supplementary Fig. S1), in bacteria the most derived (and probably the most diverged) form of CDGSH proteins as discerned in the phylogeny is a Class II CDGSH protein that also has a glutamate synthase (*Glu_synthase*) domain (Supplementary Fig. S3). In contrast, the least derived (and probably the least diverged) CDGSH protein, as identified by this analysis, is the Class II CDGSH protein with the *Fer4_19* domain (Supplementary Fig. S3). This exact form of CDGSH proteins was also identified in bacterial genomes that have their shared ancestors tracing back to 3.9 BYA (Supplementary Fig. S4). These findings suggest that the appearance of the CDGSH domain in bacteria was likely a very ancient event and resulted in the emergence and evolution of a large variety of different CDGSH proteins that are observed in the genomes of many different extant bacteria (Fig. 2).

**Figure 3:**
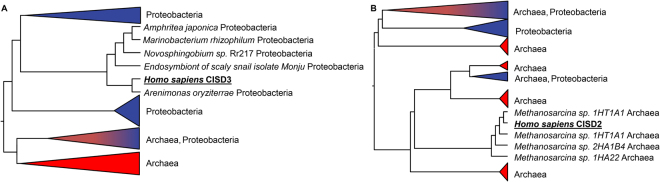
Homology of the human Class II (CISD3) and Class I (CISD2) proteins with representative archaeal and proteobacteria CISD proteins. (**A)** Phylogenetic tree of human CISD3, Archaea, and proteobacteria CISD proteins showing homology between the human CISD3 protein and proteobacteria CISD proteins. For simplification purposes, the different clades were collapsed based on the grouping of archaeal and bacterial sequences. A full version of the tree with complete protein annotations and posterior probabilities (aBayes) values is shown in Supplementary Fig. S5. (**B**) Phylogenetic tree of human CISD2, Archaea, and proteobacteria CISD proteins showing homology between the human CISD2 protein and archaeal CISD proteins. For simplification purposes, the different clades were collapsed based on the grouping of archaeal and bacterial sequences. A full version of the tree with complete protein annotations and posterior probabilities (aBayes) values is provided as Supplementary Fig. S6. Representative proteobacteria and archaea sequences were obtained as described in the Methods section. Multiple sequence alignment of the human CISD3/CISD2, archaeal, and proteobacteria CISD sequences were performed using MUSCLE and a maximum likelihood tree was generated using PhyML (see methods).

Because proteobacteria is thought to represent the bacterial taxa that originally gave rise to the mitochondria of eukaryotic cells via an endosymbiotic event^59–61^, and because the human CISD3 protein is localized to the mitochondria^5^, we constructed a phylogenetic tree of bacteria and archaea CDGSH proteins with human CISD3 (Fig. 3A, Supplementary Fig. S5). Interestingly, although archaea and eukaryotes have been reported to have diverged later than bacteria and eukaryotes^59,62^, the human mitochondrial CISD3 protein appears as a sister taxon to the proteobacterial Class II CDGSH protein and away from to the archaea Class I or Class II CDGSH proteins in the phylogenetic tree (Fig. 3A, Supplementary Fig. S5). A similar grouping of human CISD3 with proteobacterial Class II CDGSH proteins was also observed when all archaeal and bacterial CDGSH proteins were included in the phylogenetic analysis (Supplementary Fig. S6). The findings presented in Fig. 3A, Supplementary Figs S5 and S6 support the notion that the origin of the human CISD3 protein is bacterial in nature and could have emerged as a consequence of the endosymbiotic transfer event that gave rise to mitochondria.

Because archaea and eukaryotes contain Class I CDGSH proteins, whereas bacteria contain primarily the Class II CDGSH protein (Figs 1 and 2), we constructed a phylogenetic tree of bacteria and archaea CDGSH proteins with human CISD2 (a Class I CDGSH protein; Fig. 3B, Supplementary Fig. S7). As shown in Fig. 3B, human CISD2 appears to be more similar to archaeal CDGSH proteins, than to their bacterial counterparts. A similar grouping of human CISD2 with archaeal CDGSH proteins was also observed when all archaeal and bacterial CDGSH proteins were included in the phylogenetic analysis (Supplementary Fig. S8). This finding could suggest that the human Class I CDGSH proteins (represented by mNT and NAF-1) could trace their origin to an archaeal ancestor (Fig. 3B), whereas the human Class II CDGSH protein (Miner 2) could trace its origin to a bacterial one (Fig. 3A).

### Conservation of the CDGSH domain between bacteria, archaea and human

**Figure 4:**
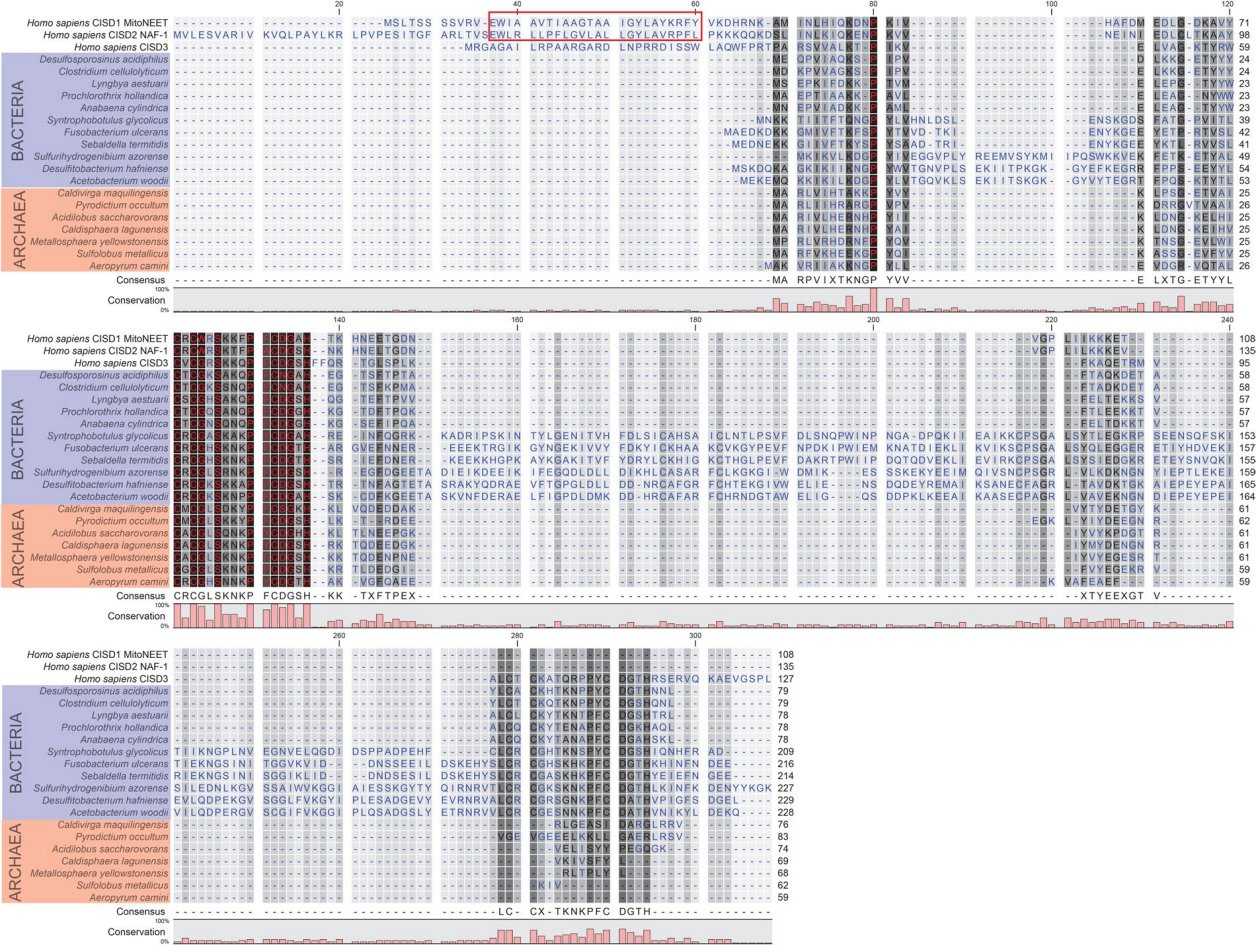
Conservation of the CDGSH domain between bacteria, archaea and human. Multiple sequence alignment analysis comparing representative CISD proteins from Aquificae, a bacterium, *Thermoprotei*, an archaeon, and human CISD1–3 proteins. Multiple sequence alignments were performed using MUSCLE. Red box indicates the transmembrane domain of CISD1 and 2. Bar graph under the aligned sequences indicates degree of conservation (%). Color legend: *Background*: White - Least conserved, Black - Most conserved; *Font*: Blue - Least conserved; Red - Most conserved. Representative bacteria and archaea sequences were obtained as described in the Methods section.

The finding of the CDGSH domain in extant genomes of organisms from all three domains of life, could potentially trace the origin of this domain to ~4 BYA when life originated (see, e.g., Class I CDGSH in *Thermoprotei*, an archaeon, Class II CDGSH in *Aquificae* a bacterium, and both types of CDGSH proteins in *Homo sapiens*; Figs 1–4, Supplementary Figs S1–S8). This intriguing possibility prompted us to assess how conserved the CDGSH domain is between these distinct prokaryotic organisms and human. We therefore performed multiple sequence alignment analysis comparing representative CDGSH proteins from *Aquificae*, *Thermoprotei*, and human. As shown in Fig. 4, the CDGSH domains of the representative archaeal and bacterial organisms chosen for this test are highly conserved with the CDGSH domains of the human CISD1–3 proteins (65% conservation; 30% identity). These findings also suggest that the regions surrounding the CDGSH iron-sulfur binding domain are highly conserved and that the canonical 3Cis-1His coordinating structure of CDGSH proteins is similar between these organisms representing different domains of life. In addition, our analysis revealed that the CDGSH domain of the Class I CDGSH proteins included in the analysis (i.e., mNT, NAF-1 and the representative archaeal sequences from *Thermoprotei*) was more similar to the CDGSH domain that is closer to the N-terminal of the Class II proteins (i.e., human CISD3 and the representative bacterial sequences from *Aquificae*), than to the CDGSH domain that is closer to the C-terminal of Class II CDGSH proteins (Fig. 4). Because the human mNT and NAF-1 proteins contain a transmembrane (TM) domain at their N-terminal^4,5,39,63^, and this domain plays an important role in their function^63^, we searched for a transmembrane domain in the *Thermoprotei* and *Aquificae* sequences. However, a TM domain could not be found in these proteins, as well as in human CISD3, suggesting that the TM domain of human CISD1/2 proteins originated later in evolution (Fig. 4).

### Molecular clock analysis of CDGSH evolution in eukaryotes

**Figure 5:**
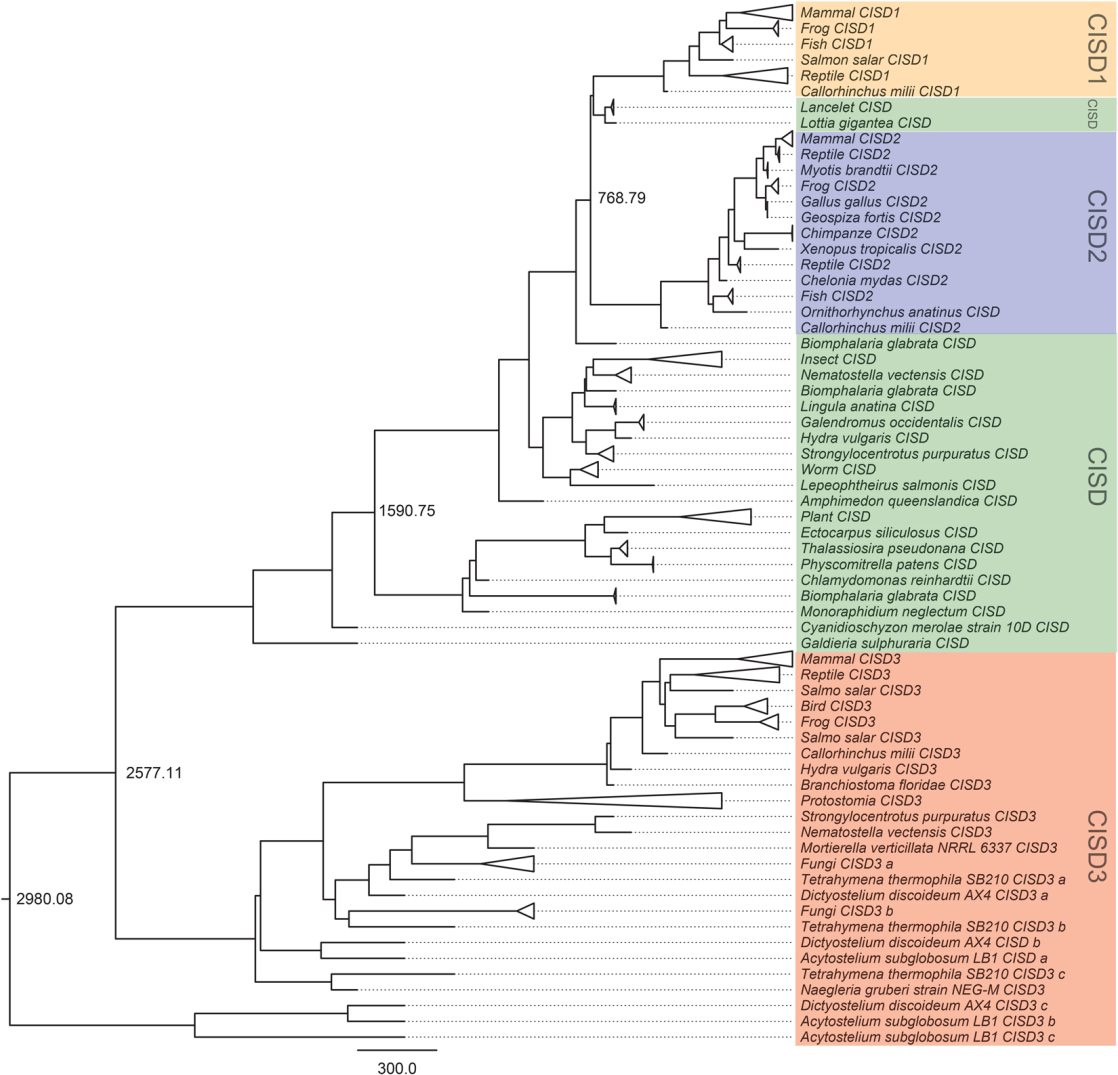
Molecular clock analysis of CDGSH evolution in eukaryotes. A time tree of divergence among eukaryotic CISD proteins constructed using the BEAST (Bayesian Evolutionary Analysis Sampling Trees) software. Eukaryotic sequences were obtained as described in the Methods section. A total of 150 sequences were subjected to Bayesian analysis using two model combinations (Supplementary Table S3. Supplementary Material) - a constant (shown here and Supplementary Fig. S9) and an exponential (Supplementary Fig. S10) population size model with a relaxed uncorrelated log-normal clock. The divergence times for Class I/II and Class III, Class I and Class II, and the putative ancestor of eukaryotic CISD proteins are shown. An expanded version of the two trees (constant and exponential) with complete protein annotations and estimated node ages is shown in Supplementary Figs S9 and S10.

Our previous analysis revealed that in eukaryotes Class I CDGSH domain proteins evolved into human NAF-1 and mNT, and Class II CDGSH proteins evolved into human CISD3^1^. However, the evolutionary timing of these events was not determined, as well as the evolutionary timing for the appearance of eukaryotic CDGSH proteins. To address these questions we used the BEAST cross-platform program for Bayesian analysis of molecular sequences using Markov Chain Monte Carlo MCMC^64–66^. Using two different models (see materials and methods section), we generated two rooted phylogenetic time trees that were very similar in their topology (Fig. 5, Supplementary Figs S9–S10). According to both trees, a distinct set of four major clades represents the eukaryotic CDGSH proteins. These include a Class II CISD3-like clade, and three Class I clades: a CISD1-like clade, a CISD2-like clade, and a clade we term CISD that contains the reminder of the Class I proteins. The CISD clade contains at least two other major sub clades with two different divergence points (Fig. 5, Supplementary Figs S9–S10). According to our analysis, the Class I and Class II eukaryotic CDGSH proteins diverged from their most recent common ancestor ~2.3–2.6 BYA, a time frame that puts this separation event at or close to the emergence of eukaryotic organisms on Earth^59^, as well as to the great oxidation event^67^. This finding could also support the notion that the progenitor of the eukaryotic Class II proteins is bacterial (Fig. 3A, Supplementary Figs S5–S6) and could have been a consequence of an endosymbiotic event that is inferred to have occurred during the early evolution of eukaryotes (Kurland and Andersson 2000; Hedges and Kumar 2009; Pittis and Gabaldón 2016). Interestingly, a sub-group of Class II CDGSH proteins from the slime molds *Dictyostelium discoideum* and *Acytostelium subglobosum* appears to precede the separation and/or endosymbiotic event that distinguished between Class I and Class II CDGSH proteins. This could suggest that multiple origins could exist for eukaryotic CDGSH proteins, potentially arising from different endosymbiotic and/or lateral gene transfer events (Fig. 5, Supplementary Figs S9–S10). The separation of animal and plant CDGSH proteins appeared to have occurred about 1.5 BYA, and the separation of Class I CISD1/2 and CISD proteins appears to have occurred 1–1.1 BYA. As previously reported^1^, plants do not contain a Class II CDGSH protein and it is possible that this class of CDGSH proteins was lost during their evolution.

The divergence of CISD1 and CISD2 from their common ancestor, that was previously postulated to coincide with the emergence of vertebrates on Earth^1^, occurred 622–768 MYA. The latter time estimate is in accordance with the tree of life timeline for the appearance of vertebrates^59^. Interestingly, in both of our trees representatives of the *Dictyostelium discoideum* and *Acytostelium subglobosum* Class I CDGSH proteins appeared within the Class II CDGSH clade (Fig. 5, Supplementary Figs S9–S10). This finding further supports the hypothesis that the slime mold CDGSH proteins could be similar in sequence to the ancient progenitor of eukaryotic CDGSH proteins^1^.

## Discussion

Our phylogenetic analysis of the CDGSH domain in prokaryotes revealed that it is highly conserved and widespread among many phyla of bacteria and archaea, suggesting that it evolved early during the emergence of life on Earth (Figs 1–4, Supplementary Figs S1–S8). Its apparent initial association with the *Fer4_19* (4Fe-4S binding) domain (Figs 1, 2, Supplementary Figs S1–S4) demonstrates strong association between the CDGSH domain and other Fe-S proteins. The finding that the 2Fe-2S CDGSH binding domain is ancient and appears in all domains of life is in agreement with the presence of high levels of iron and sulfur in the primordial oceans and the finding of many Fe-S proteins, some belonging to the *Fer4_19* family, in the inferred genome of LUCA last universal common ancestor^55–58^. In contrast to the finding of the CDGSH domain in association with the *Fer4_19* domain in bacteria and archaea (Figs 1, 2, Supplementary Figs S1-S4), we could not find the *Fer4_19* domain in eukaryotes (not shown), suggesting that some aspects of CDGSH function could be different between prokaryotes and eukaryotes.

Structural studies conducted on the human CDGSH proteins mNT, NAF-1^3,12,15,24^ and on the human and bacterial CISD3 proteins^2,68^, our previous phylogenetic analysis of eukaryotic CISD proteins^1^, and our current analysis of these proteins in archaea, bacteria and eukaryotic organisms, reveal an interesting property of CDGSH proteins. When appearing as a Class I single domain CDGSH proteins such as mNT and NAF-1, CDGSH proteins function as homodimers. In contrast, when appearing as a Class II CDGSH proteins that have two CDGSH sequences on the same polypeptide, CDGSH proteins function as a monomer. Furthermore, CDGSH proteins with 3 or more CDGSH domains on the same polypeptide were not found in our current or previous analysis of CDGSH proteins in genomes from different life domains^1^. It is therefore possible that CDGSH proteins require two CDGSH 2Fe-2S clusters in close proximity to each other to be able to function in different biological systems. Although this hypothesis, which is based on structural and phylogenetic studies, is highly speculative and would require additional structural and evolutionary studies to be validated, it nevertheless bears importance when attempting to speculate on the origins and functions of ancient CDGSH proteins. Did these proteins originate as a Class I single domain, or did they originate as a Class II double domain? Although we may never know the answer to this question, our findings that bacteria primarily contain Class II CDGSH domain proteins, and that the ancient archetype of CDGSH proteins in bacteria could potentially be a Class II protein associated with a *Fer4_19* domain (Fig. 2, Supplementary Figs S3, S4), suggest that the Class II domain organization structure might have an initial evolutionary advantage, explaining its retention in many eukaryotic organisms and bacteria. Of course, to generate a double CDGSH domain Class II protein, an initial duplication event of a single domain was required. In this respect it should be noted that, of the two CDGSH domains of Class II proteins, the CDGSH domain closer to the N-terminal of these proteins appears to have a higher degree of homology to the CDGSH domain of Class I proteins (Fig. 4), suggesting that the Class I proteins could have emerged after a deletion of the of the distal part of the ancient Class II gene encoding C-terminal CDGSH domain, or that the ancient Class II CDGSH proteins emerged after a duplication of the sequence encoding the N-terminal CDGSH domain in an ancient Class I gene.

Our findings that proteobacterial Class II CDGSH proteins are more similar to human and slime mold CISD3 proteins than to archaeal CDGSH proteins (Fig. 3A, Supplementary Figs S5–S6), suggest that human and perhaps other eukaryotic CISD3 proteins could trace their origin to the ancient proteobacterial genome that gave rise to mitochondria through the endosymbiotic transfer event. In contrast, the finding that the human Class I CISD2 protein is more closely related to archaeal Class I and II CDGSH proteins than to proteobacterial Class II CDGSH proteins (Fig. 3B, Supplementary Figs S6–S8) suggests that the ancestor of human Class I CDGSHs protein evolved after the radiation of bacteria and archaea/eukaryotes. If this hypothesis holds true, then the origins of eukaryotic Class I single domain proteins could be distinct from that of eukaryotic Class II proteins (Fig. 3, Supplementary Figs S5–S8). Further studies are required to address this possibility. Additionally, our phylogenetic analysis revealed some interesting instances of possible horizontal gene transfer between archaea and bacteria. For example, CDGSH containing protein sequence of *Asticcacaulis benevestitus* (Proteobacteria, WP_018079727.1) was embedded within an archaeal clade, with *Halobaculum gomorrense* (Archaea, WP_073307495.1) as the nearest neighbor (Supplementary Fig. S6). This clade had a bootstrap confidence of 94.36%, providing support to the possibility of inter-domain gene transfer from an archaeon (*Halobaculum gomorrense*) to a bacterium (*Asticcacaulis benevestitus*). Furthermore, as both these strains are aquatic isolates and dwell in hypersaline environment, their shared ecology might have facilitated gene exchange including of those harboring CDGSH domains^69,70^.

**Figure 6:**
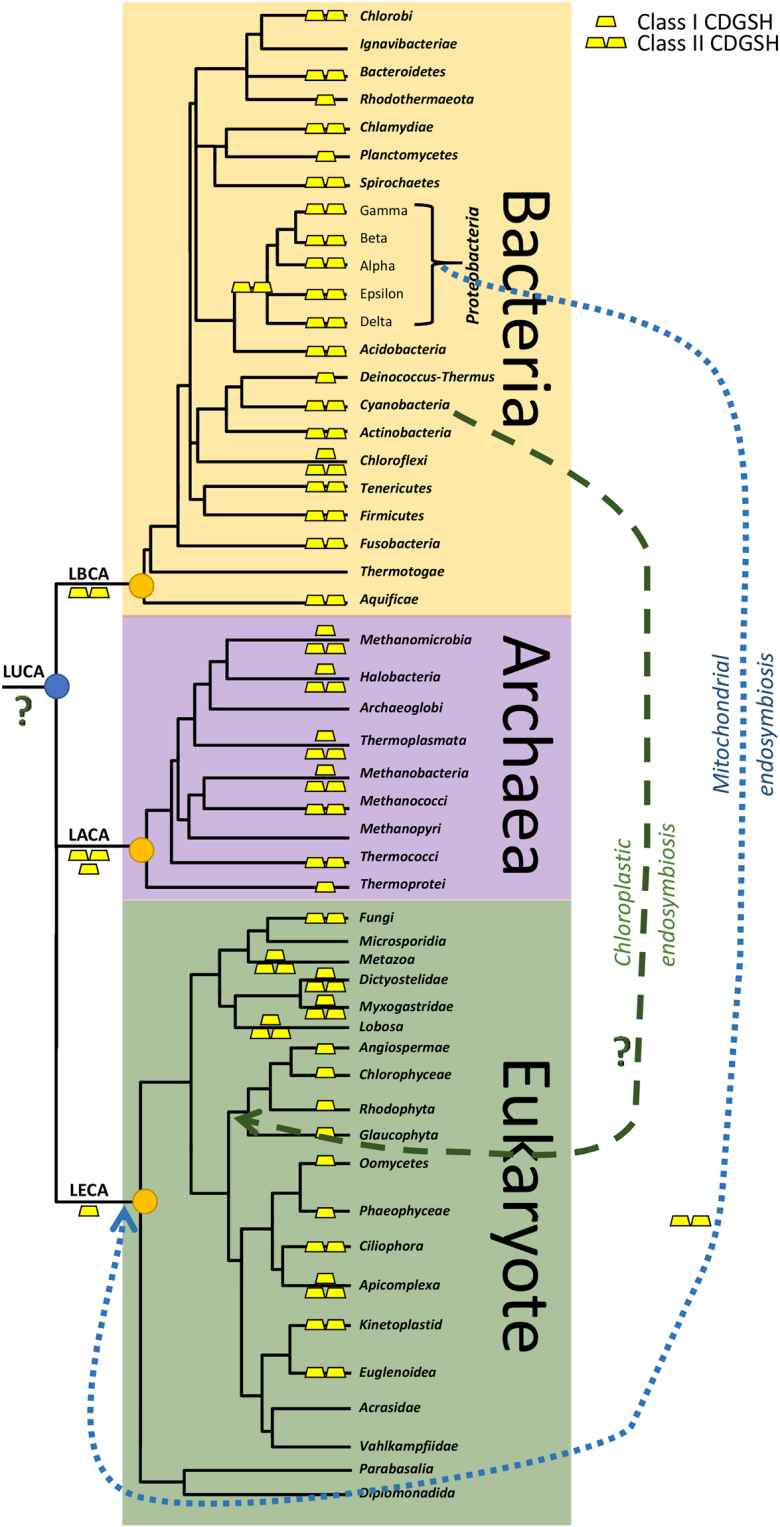
A hypothetical model for the evolution of CDGSH proteins. The occurrence of Class I and Class II CDGSH proteins is shown in major lineages from the 3 domains of life. Bacteria are shown to primarily contain Class II CDGSH proteins, whereas archaea and eukaryotes contain Class I and Class II CDGSH proteins. Eukaryote Class II CDGSH proteins are proposed to have originated from the endosymbayotic event that yielded the mitochondria. Class I CDGSH proteins are postulated to have evolved only after archaea and eukaryotes have radiated from bacteria. LUCA, last universal common ancestor, LACA, last archaeal common ancestor, LECA, last eukaryote common ancestor, LBCA, last bacterial common ancestor.

The evolutionary trajectory of the CDGSH domain is proposed in Fig. 6. In this model, it is hypothesized that a prototype of Class II CDGSH protein is the last common ancestor of all CDGSH proteins. This Class II double domain protein originated from an early duplication event and was retained in the genomes of representative organisms from all domains. As speculated above, this type of CDGSH protein (Class II) provided an adaptive advantage owing to its role in Fe-S driven reactions in early organisms and therefore the archetypal Class II CDGSH gene was selected for and retained in the course of evolution, and the genomes of almost all extant organisms from bacteria to archaea to eukaryotes harbor the Class II CDGSH gene. The appearance of the Class I single domain CDGSH protein that has been reported to function as a homodimer^3,12,15,24^ might have independently occurred after bacterial and archaeal/eukaryotic lineages diverged from their common ancestor, and is currently found primarily in archaea and eukaryotes. Because many of the Class II CDGSH proteins of both bacteria and archaea contain the *Fer4_19* domain, but eukaryotic Class II CDGSH proteins do not, it is possible that archaea and bacteria Class II proteins are related. In contrast, all eukaryotic Class II CDGSH proteins could have evolved from an ancient proteobacteria Class II CDGSH protein that might have lost the *Fer4_19* domain or more likely, this domain got lost after the primary endosymbiotic gene transfer event. It is also possible that once Class I CDGSH proteins evolved, some organisms, for example plants, or certain bacterial and archaeal lineages, lost the Class II domain protein and retained only the Class I CDGSH protein. The model described above suggest that the evolution of a two domain CDGSH protein via domain duplication preceded the evolution of the single domain CDGSH protein that requires a homodimeric structure to function. Because a simple domain duplication rather than the emergence of mechanisms for two identical proteins to dimerize (likely to require a stepwise evolutionary process involving changes to many different amino acids at the surface of the protein) appears more parsimonious and thus plausible. It is reasonable to speculate that the function of the Class II proteins was initially established through domain duplication. This event was then followed by the more complex process of Class I homodimer protein evolution (a single domain protein that could function as a homodimer). Once this new form of protein (Class I homodimer) was established, the Class II protein could have been lost in some lineages, as is likely the case in plants^1^. The high prevalence of proteins containing the CDGSH domain in bacteria and archaea is similar to that of proteins containing other important domains such as the catalase heme or the Fer4_19 domains, indicating that the CDGSH domain could have played an important role in evolution (Supplementary Figs S11, S12). Further studies are therefore required to address the origin and function of this fascinating and highly conserved CDGSH iron-sulfur binding motif.

## Methods

### Selection of organisms for analysis

For eukaryotes, we selected representative organisms from different lineages with fully sequenced and annotated genomes as described in^1^. Briefly, human CISD1, CISD2, and CISD3 were used as query sequences to perform a PSI-BLAST search to obtain the CISD homologs from the genomes of the organisms selected for our analysis^71^. The default parameter setting of PSI-BLAST was used, with Expect threshold of 10 and PSI-BLAST threshold of 5. A total of 150 sequences were selected and the multiple sequence alignments were analyzed using BEAST (Bayesian Evolutionary Analysis Sampling Trees) for determining the divergence time of the CISD genes^65,66^. As previous analysis had reported *Dictyostelium discoideum* (a protist slime mold unicellular cell) as the possible representative of the most ancient CISD gene in eukaryotes^1^, we used the *Dictyostelium* CISD (XP_647247.1) sequence as the query sequence to search for potential bacterial CISD homologs in the non-redundant database using PSI-BLAST^71^. PSI-BLAST iterations were performed until no new BLAST hit was retrieved. All sequences thus obtained were subjected to domain analysis using PFAM, which utilizes profile hidden Markov model to predict the domain architecture of the protein sequences^72^. Sequences with at least one CDGSH domains were kept, and incomplete and partial sequences were discarded. A total of 494 bacterial sequences representing different lineages were selected for further analysis (Supplementary Table S1). In order to retrieve CISD homologs in archaea, we performed a PSI-BLAST search against the non-redundant database using the same *Dictyostelium* CISD sequence as the query. All sequences were again subjected to domain analysis using PFAM and sequences possessing at least one CDGSH domains were retained. A total of 191 archaeal sequences representing different lineages were selected for further analysis (Supplementary Table S2).

### Sequence alignment

For the above three sets representing bacterial, archaeal, and eukaryotic CISD sequences, multiple sequence alignment was performed for each using command-line multiple alignment program MUSCLE with default options^73^. trimAL was used (-automated1 option) to remove poorly aligned regions in order to obtain high quality alignments^74^.

### Divergence time estimation for eukaryotic sequences

Multiple sequence alignments of eukaryotic CISD sequences were analyzed using BEAST for the estimation of divergence times. Multiple combinations of population size change and molecular clock models were assessed in order to find the best-fit model. Among the models tested, the combination of a constant/exponential population size model and a relaxed uncorrelated log-normal clock with high estimated sample size (ESS) yielded the highest Bayes factor (Supplementary Table S3). Both selected models allowed the evolutionary rates to change among the branches of the tree and had the BLOSUM62 substitution model with γ correction for among-site rate variations^64^. The time calibration points for each organism were obtained from the TimeTree website www.timetree.org^59^.

All BEAST Monte Carlo Markov Chain (MCMC) simulations were run for at least 50 million steps, with subsampling at every 1,000 steps. The trees generated by BEAST were summarized by a single maximum clade credibility (MCC) tree using TreeAnnotator^64^ with 20% of the MCMC steps discarded as burn-ins. Statistical uncertainty is represented by a 95% confidence interval (CI) calculated as the 95% highest posterior density (HPD) interval (upper-lower). The final MCC tree was visualized and edited with the program FigTree (http://tree.bio.ed.ac.uk/software/figtree/)^75^. The inferred time of divergence from an ancestral node is indicated next to each internal node in Fig. 5, Supplementary Figs S9–S10).

### Maximum likelihood phylogenetic analysis of bacterial and archaeal sequences

PhyML version 3.0 was utilized to generate maximum-likelihood trees for bacterial and archaeal CISD sequences^76^. For statistical reliability, the following tests were used: an approximate likelihood-ratio test (aLRT) based on logarithm of the ratio of likelihood computed for the current tree and that of the best alternative, and a Bayesian-like transformation of aLRT (aBayes). To estimate the optimal model of substitution, ProtTest was used for each alignment^77^. ProtTest indicated the WAG amino acid model with gamma distribution shape parameter (WAG + G) and the WAG amino acid model with invariable gamma distribution shape parameter (WAG + I + G) as the best fitting models among the 112 examined evolutionary models, based on Akaike information criterion (AIC) statistics, for archaea and bacteria respectively. The trees were visualized and designed with iTOL (Interactive Tree of Life) web-server^78^. The domain organization for each sequence was appended at the end of terminal branches, using iTOL, as shown in Supplementary Figs S1–S4.

### Time-tree of organisms

To generate an evolutionary timescale for the bacterial and archaeal organisms represented in our analysis, we generated time-trees using TimeTree.org website. The complete lists of representative bacterial and archaeal organisms (Tables S1 and S2) were uploaded separately to generate a time-tree for each. The time-tree represents the estimated divergence time between species or groups of species or lineages based on literature records^59^. However, the divergence times for multiple bacterial and archaeal organisms were not found in the TimeTree website. Nevertheless, we ensured that at least one organism from a class or phylum is represented in the time-tree.

### Data availability statement

All data used in this study is publically available. All data or tools generated by this study will be made available upon request.

## Electronic supplementary material

Supplementary Material
