# Supplementary material for: Phylogenetic analysis of the CDGSH iron-sulfur binding domain reveals its ancient origin
Source: Sci Rep. 2018 Mar 19;8:4840. doi: 10.1038/s41598-018-23305-6 (PMC5859297; doi:10.1038/s41598-018-23305-6)
Supplement: Supplementary file 1 — Supplementary Material [file 41598_2018_23305_MOESM1_ESM.pdf]

Supplementary Material for:

Phylogenetic analysis of the CDGSH iron-sulfur binding domain reveals it ancient origin

Soham Sengupta[1], Rachel Nechushtai[2], Patricia A. Jennings[3], Jose' N. Onuchic[4], Pamela Padilla[1], Rajeev K. Azad[1,5] and Ron Mittler[1,*]

[1]Department of Biological Sciences, University of North Texas, Denton TX 76203, USA. [2]The Alexander Silberman Institute of Life Science, Hebrew University of Jerusalem, Edmond J. Safra Campus at Givat Ram, Jerusalem 91904, Israel. [3]Department of Chemistry & Biochemistry, University of California at San Diego, La Jolla, CA 92093, USA. [4]Center for Theoretical Biological Physics and Department of Physics, 239 Brockman Hall, 6100 Main Street- MS-61, Rice University, Houston, TX 77005, USA. [5]Department of Mathematics, University of North Texas, Denton, TX 76203, USA.

* To whom correspondence should be addressed: ronmittler@gmail.com; Tel: 1-940-293-7170

Supplementary Figure S1: Maximum likelihood tree of archaeal CISD sequences generated using PhyML.

Supplementary Figure S2: Time-tree of archaea. The time of divergence of the archaeal organisms were obtained from the TimeTree.org. The domain organization for each organism was appended at the end using iTOL (https://itol.embl.de/).

Supplementary Figure S3: Maximum likelihood tree of bacterial CISD sequences generated using PhyML.

Supplementary Figure S4: Time-tree of bacteria. The time of divergence of the archaeal organisms were obtained from the TimeTree.org. The domain organization for each organism was appended at the end using iTOL (https://itol.embl.de/).

Supplementary Figure S5: Extended maximum likelihood tree of human CISD3, Archaea, and proteobacteria generated using PhyML tree.

Supplementary Figure S6: Maximum likelihood tree of human CISD3, archaea, and bacteria generated using PhyML.

Supplementary Figure S7: Extended maximum likelihood tree of human CISD2, archaea, and proteobacteria generated using PhyML.

Supplementary Figure S8: Maximum likelihood tree of human CISD2, archaea, and bacteria generated using PhyML.

Supplementary Figure S9: Extended time tree of divergence of eukaryotic CISD sequences generated using BEAST. The model was selected to be a combination of exponential population size model with a relaxed uncorrelated log-normal clock.

Supplementary Figure S10: Time tree of divergence of eukaryotic CISD sequences generated using BEAST. The model was selected to be a combination of constant population size model with a relaxed uncorrelated log-normal clock.

Supplementary Figure S11: Occurrence of the CDGSH, Fer4_19 and Heme (catalase) domains in archaea. A time tree for all archaea classes was obtained from TimeTree.org. The presence or absence of the three domains within each class was determined using the *Dictyostelium* CISD (XP_647247.1) (for CDGSH), the Fer4_19 (PF06902), and the *Methanolacinia paynteri* (WP_048151059.1) catalase sequence as the query sequence to perform PSIBLAST. Node ages are represented in billion years ago (BYA).

Supplementary Figure S12: Occurrence of the CDGSH, Fer4_19 and Heme (catalase) domains in Bacteria. A time tree for all bacteria phylum was obtained from TimeTree.org. The presence or absence of the three domains within each class was determined using the *Dictyostelium* CISD (XP_647247.1) (for CDGSH), the Fer4_19 (PF06902), and the *Rufibacter ruber* (WP_066836067.1) catalase sequences as the query sequence to perform PSI-BLAST. Node ages are represented in billion years ago (BYA).

Supplementary Table S1: List of bacterial CISD sequences

Supplementary Table S2: List of archaeal CISD sequences

Supplementary Table S3: BEAST model test table.

Supplementary Figure S1: Maximum likelihood tree of archaeal CISD sequences generated using PhyML.

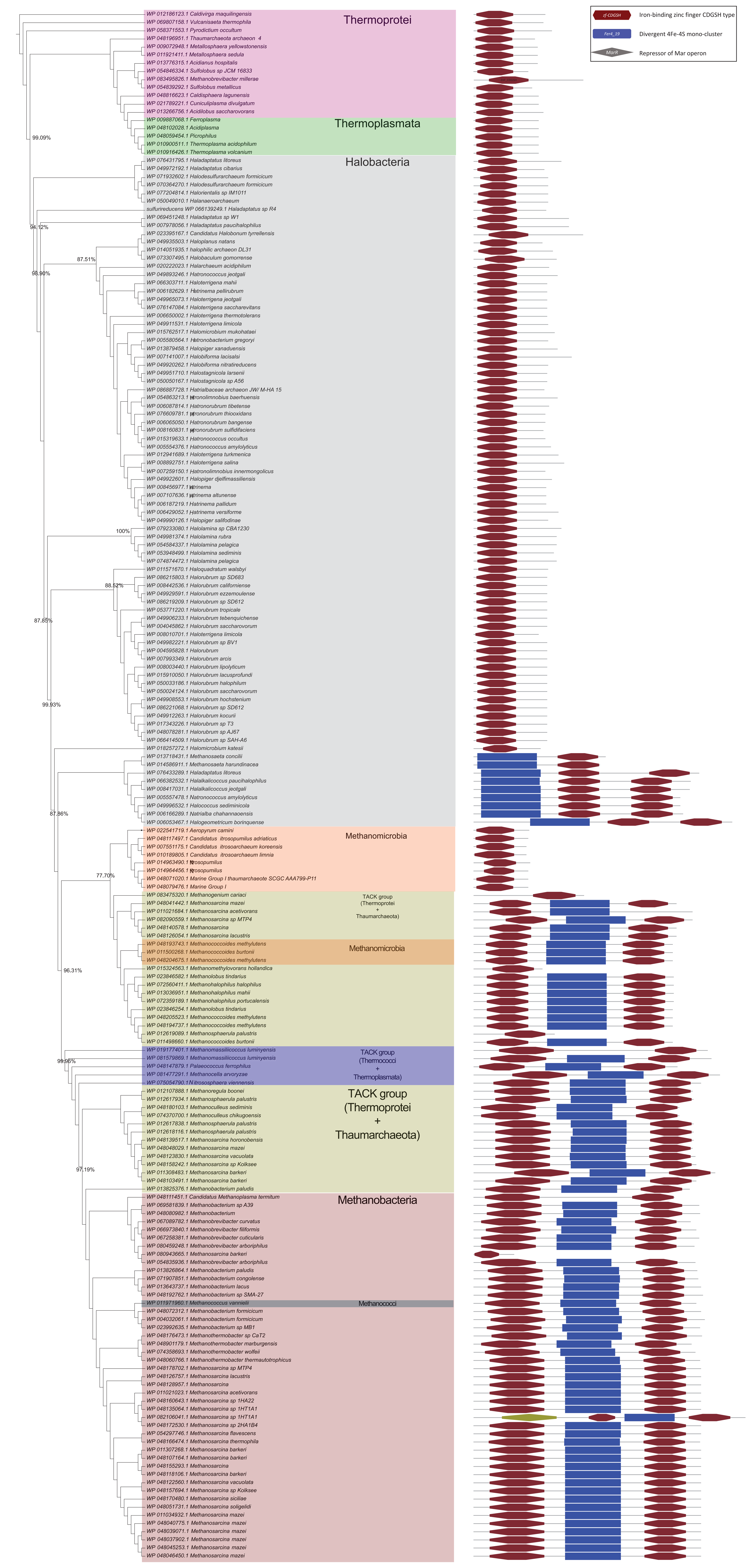

Supplementary Figure S2: Time-tree of archaea. The time of divergence of the archaeal organisms were obtained from the TimeTree.org. The domain organization for each organism was appended at the end usin iTOL (https://itol.embl.de/).

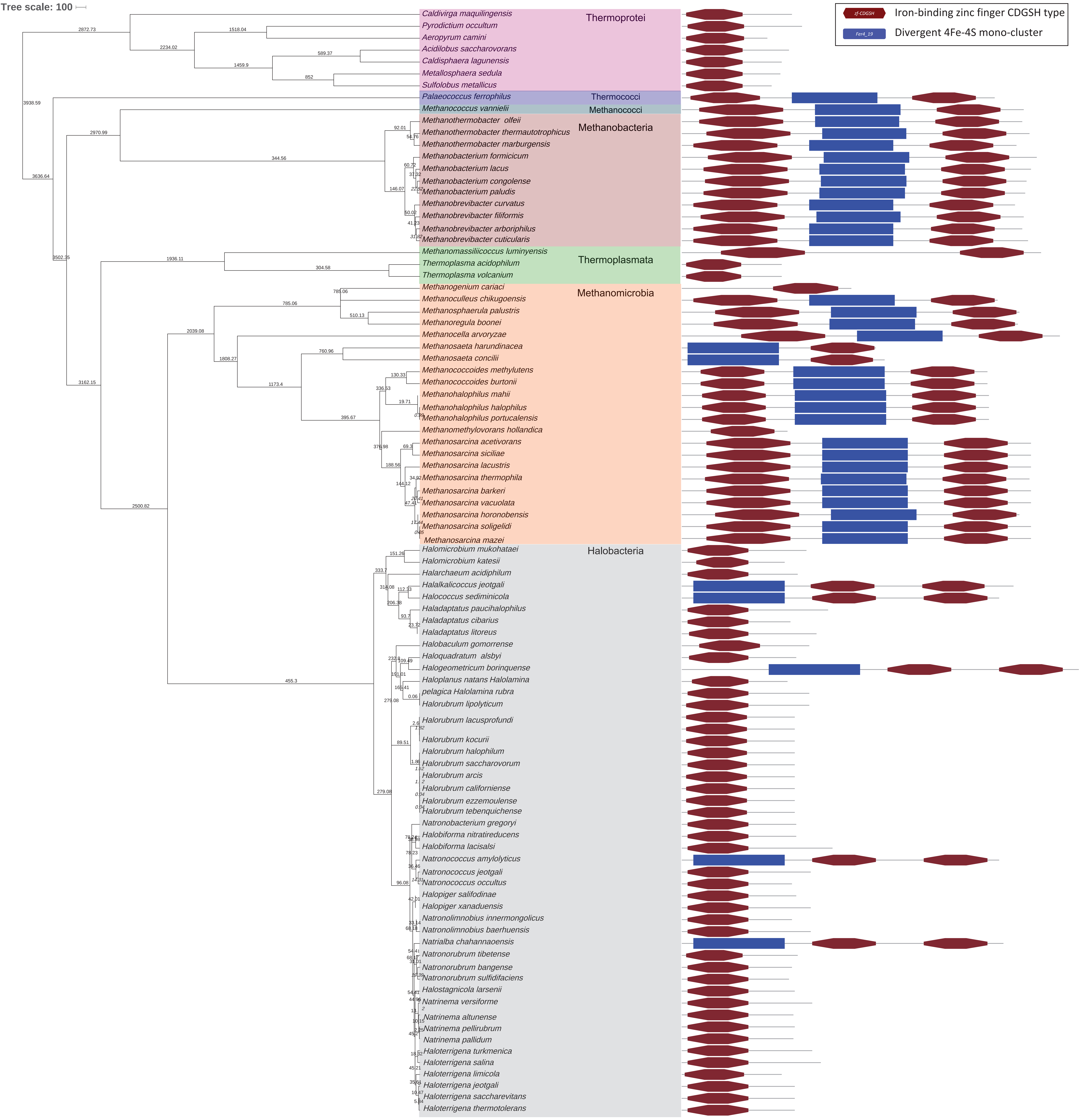

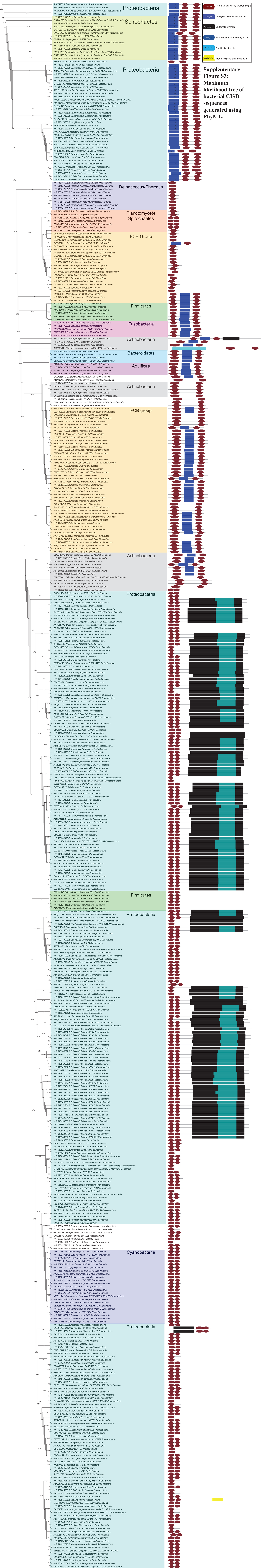

Supplementary Figure S3: Maximum likelihood tree of bacterial CISD sequences generated using PhyML.

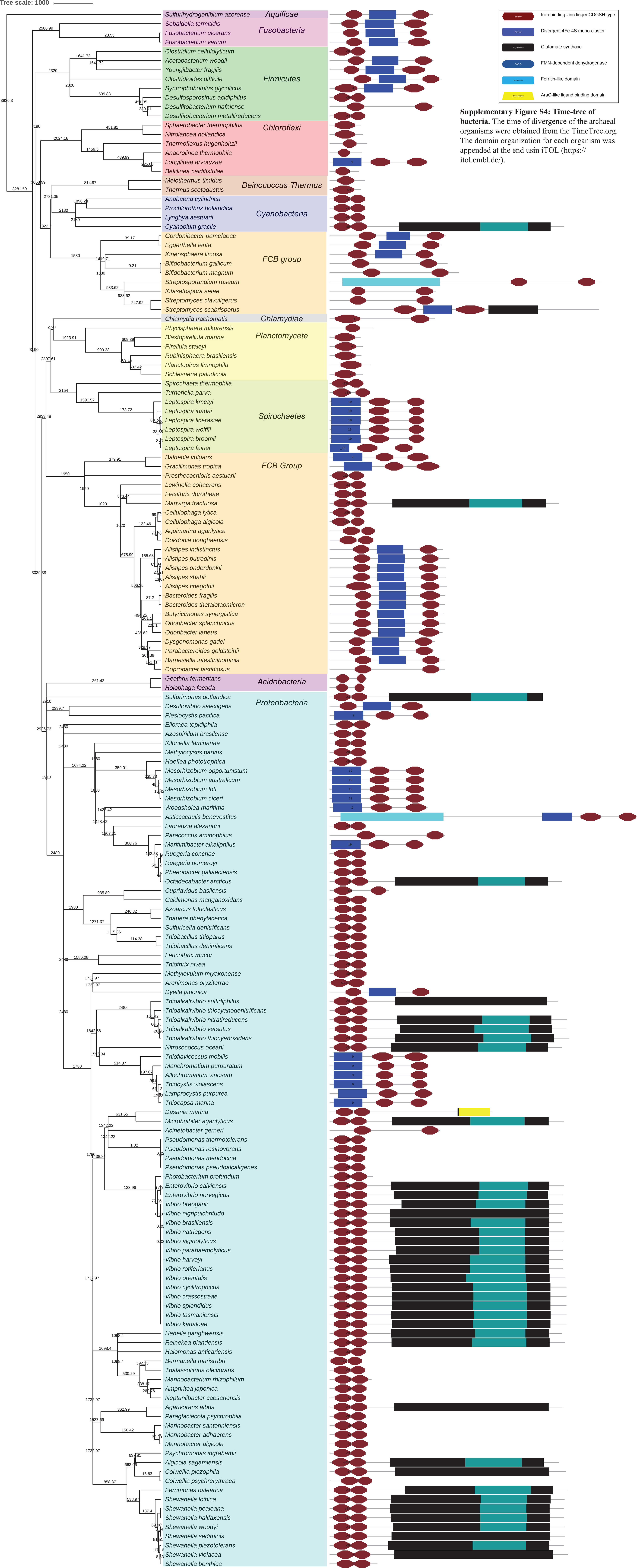

Supplementary Figure S4: Time-tree of bacteria. The time of divergence of the archaeal organisms were obtained from the TimeTree.org. The domain organization for each organism was appended at the end usin iTOL (https://itol.embl.de/).

Supplementary Figure S5: Extended maximum likelihood tree of human CISD3, Archaea, and proteobacteria generated using PhyML tree.

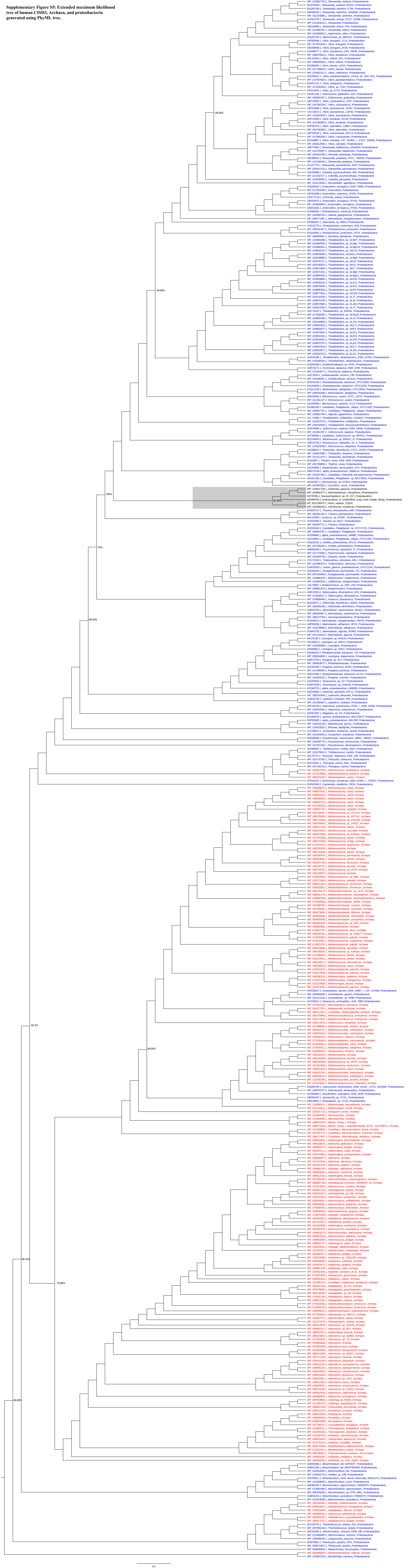

Supplementary Figure S6: Maximum likelihood tree of human CISD3, archaea, and bacteria generated using PhyML.

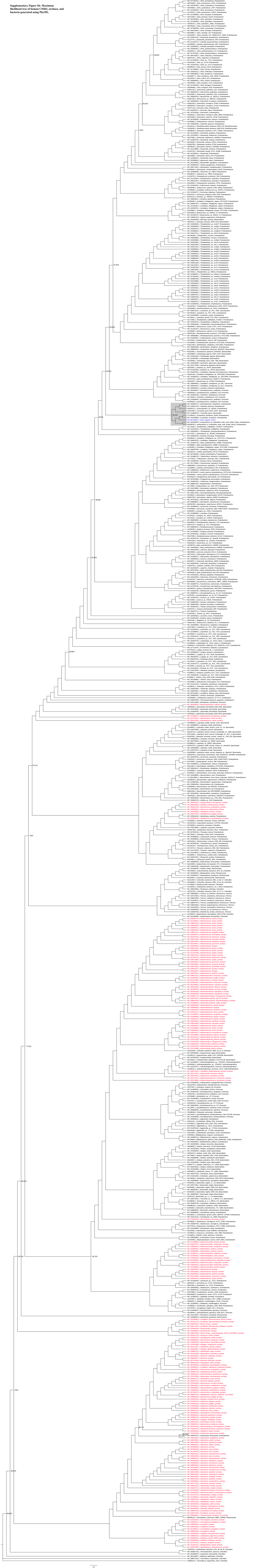

Supplementary Figure S7: Extended maximum likelihood tree of human CISD2, archaea, and proteobacteria generated using PhyML.

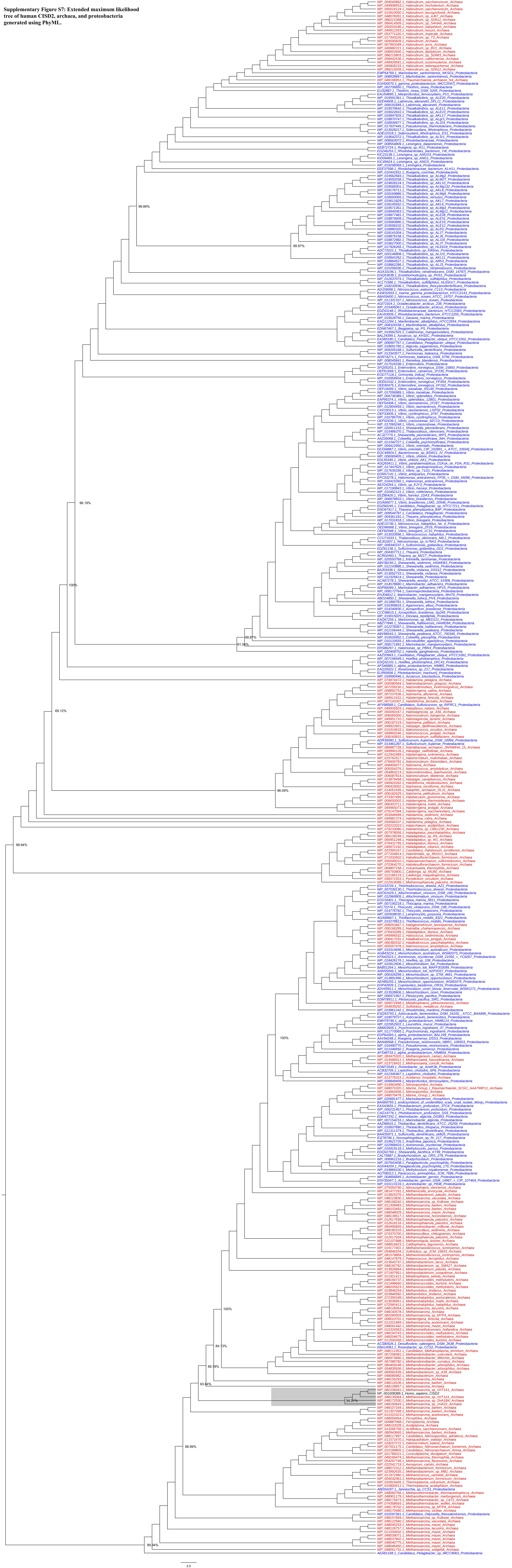

Supplementary Figure S8: Maximum likelihood tree of human CISD2, archaea, and bacteria generated using PhyML.

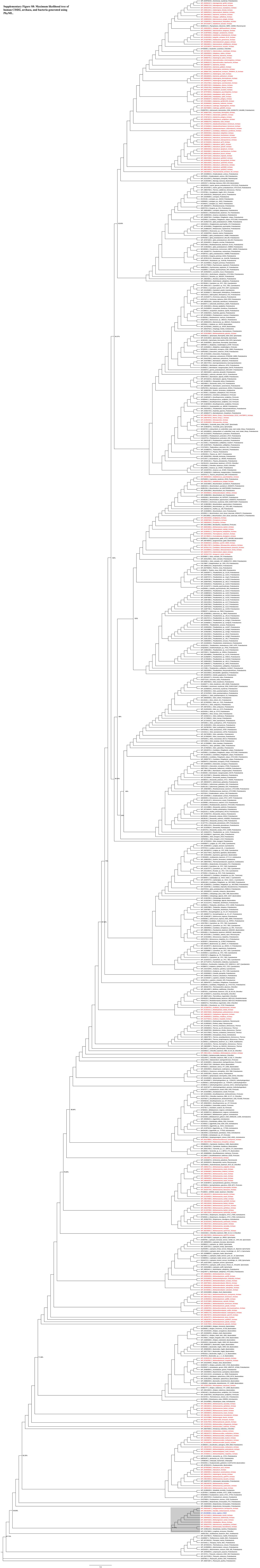

Supplementary Figure S9: Extended time tree of divergence of eukaryotic CISD sequences generated using BEAST. The model was selected to be a combination of exponential population size model with a relaxed uncorrelated log-normal clock.

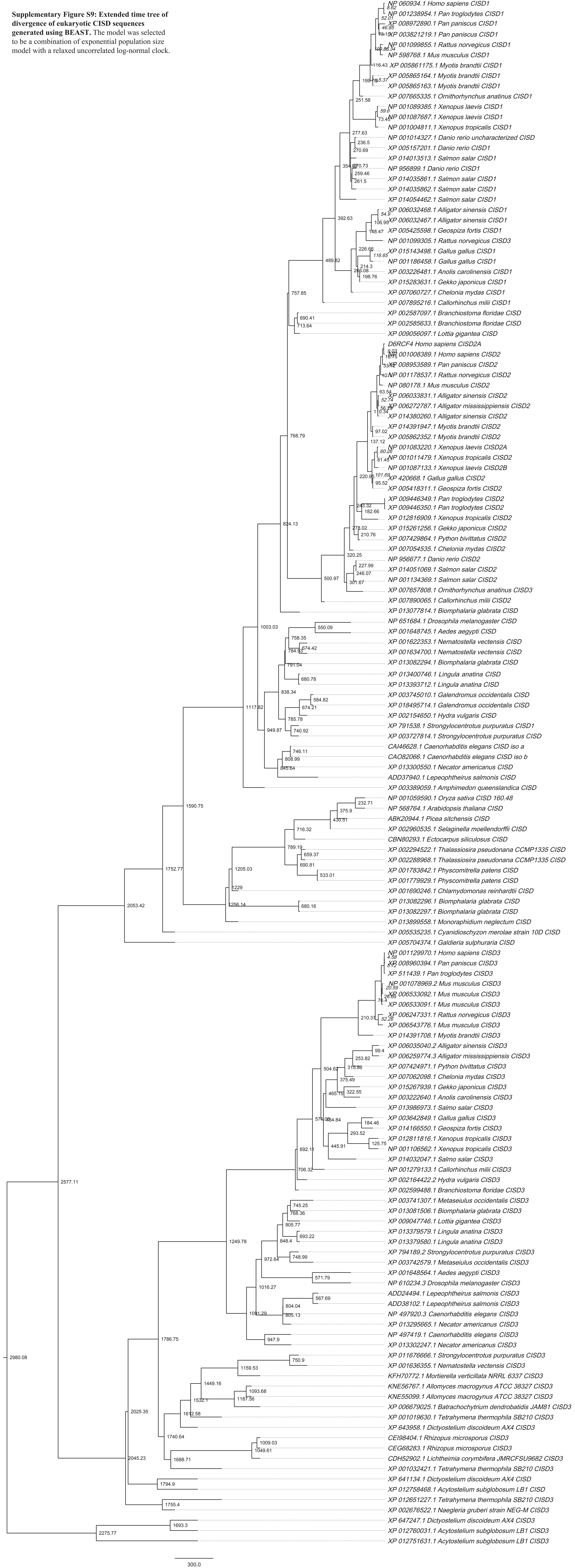

Supplementary Figure S10: Time tree of divergence of eukaryotic CISD sequences generated using BEAST. The model was selected to be a combination of constant population size model with a relaxed uncorrelated log-normal clock.

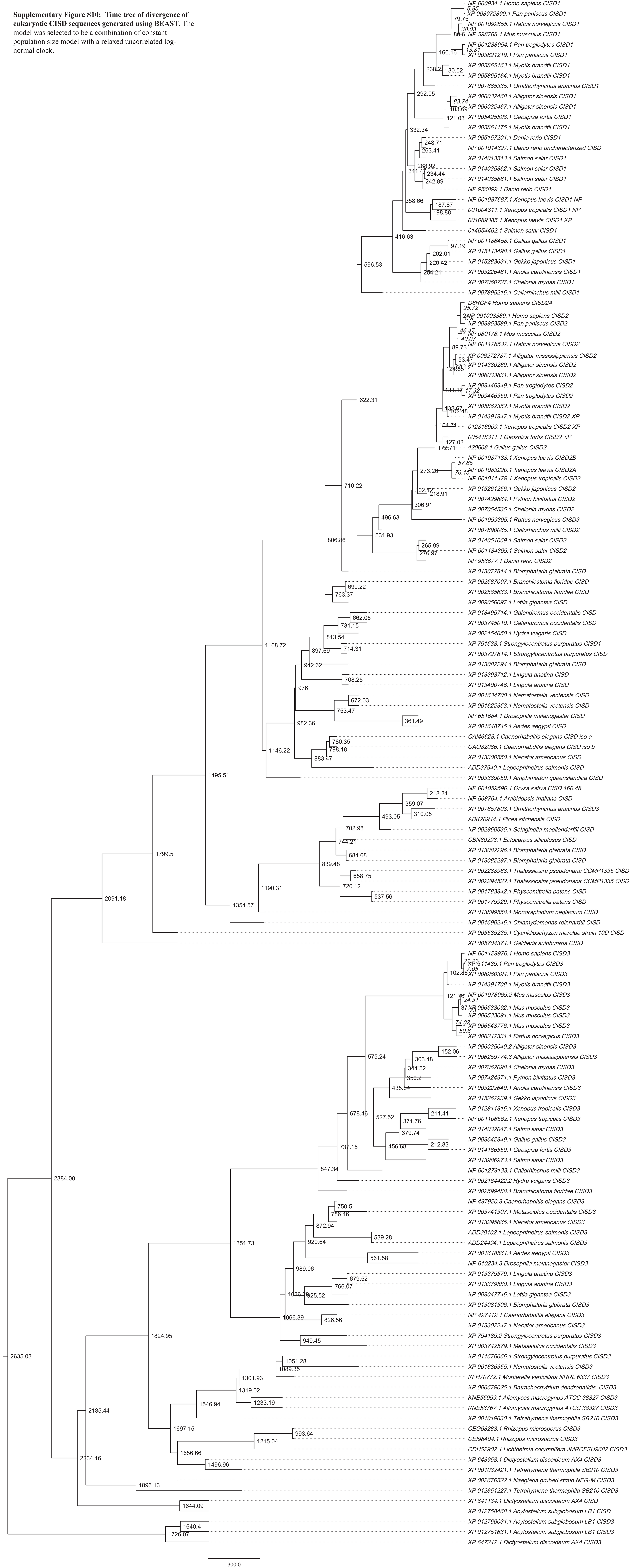

Supplementary Figure S11: Occurrence of the CDGSH, Fer4_19 and Heme (catalase) domains in archaea.

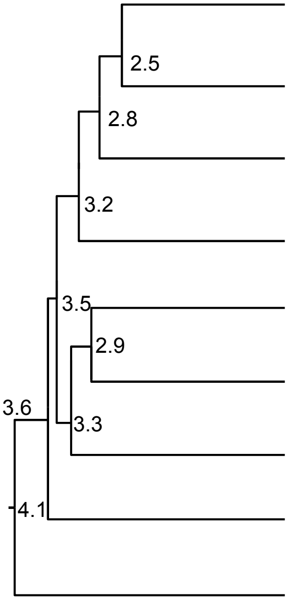

	CDGSH	Fer4_19	Heme (Catalase)
Methanomicrobia	✓	✓	✓
Halobacteria	✓	✓	✓
**Archaeoglobi*	✗	✗	✗
Thermoplasmata	✓	✗	✓
Methanobacteria	✓	✓	✓
Methanococci	✓	✓	✗
**Methanopyri*	✗	✗	✗
Thermococci	✓	✓	✗
Thermoprotei	✓	✗	✗

Supplementary Figure S12: Occurrence of the CDGSH, Fer4_19 and Heme (catalase) domains in Bacteria.

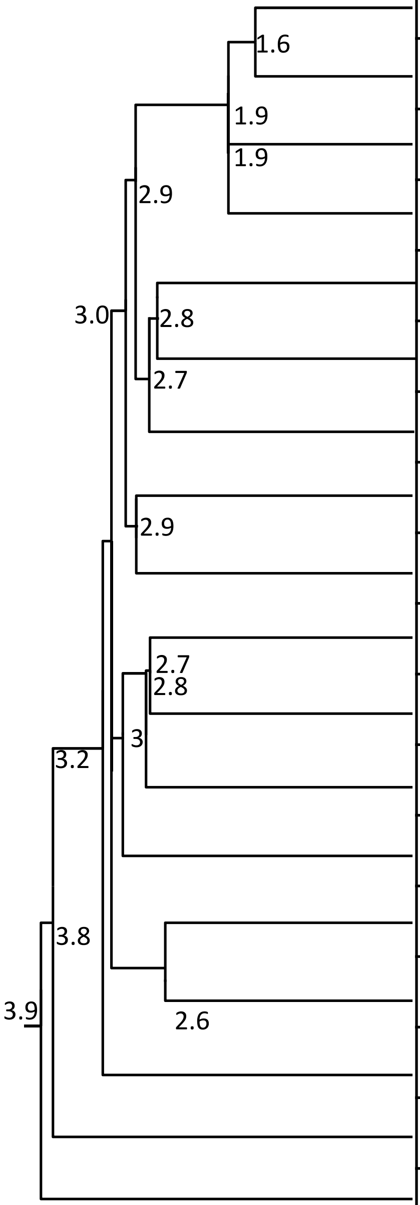

	CDGSH	Fer4_19	Heme (Catalase)
Chlorobi	✓	✓	✓
**Ignavibacteriae*	✗	✗	✓
Bacteroidetes	✓	✓	✓
Rhodothermaeota	✓	✗	✓
Chlamydiae	✓	✗	✓
Planctomycetes	✓	✗	✓
Spirochaetes	✓	✓	✓
Proteobacteria	✓	✓	✓
Acidobacteria	✓	✓	✓
Deinococcus-Thermus	✓	✗	✓
Cyanobacteria	✓	✗	✓
Actinobacteria	✓	✗	✓
Chloroflexi	✓	✓	✓
Tenericutes	✓	✗	✓
Firmicutes1	✓	✓	✓
Fusobacteria	✓	✓	✗
**Thermotogae*	✗	✗	✓
Aquificae	✓	✓	✗

Supplementary Table S1: List of bacterial CISD sequences

Bacterial CDGSH sequences	
**Accession no.**	**Organism Name**
AFA47377.1	Acetobacterium woodii
WP_14354980.1	Acetobacterium woodii
ANM31758.1	Acidobacteria bacterium
OLC84025.1	Acidobacteria bacterium
OYW04665.1	Acidobacteria bacterium
ENV35047.1	Acinetobacter gerneri
WP_4856845.1	Acinetobacter gerneri
WP_10113133.1	Acinetobacter sp.
WP_16399816.1	Agarivorans albus
WP_18691760.1	Algicola sagamiensis
AFL79483.1	Alistipes finegoldii
WP_19244998.1	Alistipes ihumii
EHB91777.1	Alistipes indistinctus
WP_9134632.1	Alistipes indistinctus
WP_19129448.1	Alistipes obesi
WP_18695858.1	Alistipes onderdonkii
EDS04537.1	Alistipes putredinis
WP_19150168.1	Alistipes senegalensis
CBK63274.1	Alistipes shahii
WP_15546209.1	Alistipes shahii
SDZ99085.1	Alistipes timonensis
WP_10259380.1	Alistipes timonensis
ABR49871.1	Alkaliphilus metalliredigens
WP_12064831.1	Alkaliphilus metalliredigens
ADC61629.1	Allochromatium vinosum
WP_12969905.1	Allochromatium vinosum
AFS46885.1	alpha proteobacterium
AFS48733.1	alpha proteobacterium
EDP64383.1	alpha proteobacterium
EMH79746.1	alpha proteobacterium
WP_7674265.1	alpha proteobacterium
WP_14952718.1	alpha proteobacterium
WP_14954564.1	alpha proteobacterium
WP_19621726.1	Amphritea japonica
WP_19622026.1	Amphritea japonica
AFZ58673.1	Anabaena cylindrica
WP_15215299.1	Anabaena cylindrica
WP_16949416.1	Anabaena sp.
WP_13560237.1	Anaerolinea thermophila
OQY33406.1	Anaerolineaceae bacterium
OIO87915.1	Anaerolineae bacterium
OQY81419.1	Anaerolineae bacterium
WP_10177493.1	Aquimarina agarilytica
WP_10522298.1	Aquimarina agarivorans

KFN42523.1	Arenimonas oryziterrae
KFN43568.1	Arenimonas oryziterrae
WP_22968433.1	Arenimonas oryziterrae
WP_22970218.1	Arenimonas oryziterrae
ESQ93793.1	Asticcacaulis benevestitus
WP_18079727.1	Asticcacaulis benevestitus
BAL24399.1	Azoarcus sp.
WP_15435704.1	Azoarcus sp.
WP_18990446.1	Azoarcus toluclasticus
WP_18991539.1	Azoarcus toluclasticus
CCC98615.1	Azospirillum brasilense
WP_14240830.1	Azospirillum brasilense
EQC49924.1	Bacteriovorax sp.
WP_21269747.1	Bacteriovorax sp.
EFR53310.1	Bacteroides fragilis
EKA82082.1	Bacteroides fragilis
EKA91819.1	Bacteroides fragilis
WP_5777921.1	Bacteroides fragilis
WP_5806300.1	Bacteroides fragilis
WP_5820357.1	Bacteroides fragilis
EFI04753.1	Bacteroides sp.
EJZ64282.1	Barnesiella intestinihominis
WP_8862003.1	Barnesiella intestinihominis
EDN67467.1	Beggiatoa sp.
WP_82149097.1	Bellilinea caldifistulae
EFA23548.1	Bifidobacterium gallicum
WP_6294042.1	Bifidobacterium gallicum
KFI69336.1	Bifidobacterium magnum
WP_22859714.1	Bifidobacterium magnum
WP_2655533.1	Blastopirellula marina
WP_8961216.1	Bradyrhizobium Proteobacteria
CAL75887.1	Bradyrhizobium sp.
WP_19119366.1	Brevibacillus massiliensis
WP_18336656.1	Butyricimonas synergistica
WP_19562325.1	Caldimonas manganoxidans
WP_38469593.1	Candidatus Izimaplasma
WP_95544437.1	Candidatus Izimaplasma
WP_7221991.1	Candidatus Jettenia
WP_10297361.1	Candidatus Odyssella
AAZ20963.1	Candidatus Pelagibacter
AAZ20965.1	Candidatus Pelagibacter
AEA81168.1	Candidatus Pelagibacter
EAS85180.1	Candidatus Pelagibacter
EDZ60245.1	Candidatus Pelagibacter
WP_6997767.1	Candidatus Pelagibacter
WP_6997769.1	Candidatus Pelagibacter
WP_8544787.1	Candidatus Pelagibacter

WP_11281501.1	Candidatus Pelagibacter
WP_13695333.1	Candidatus Pelagibacter
WP_20169731.1	Candidatus Pelagibacter
AFV98568.1	Candidatus Sulfuricurvum
ADV50889.1	Cellulophaga algicola
WP_13552340.1	Cellulophaga algicola
WP_13622581.1	Cellulophaga Bacteroidetes
ADY30838.1	Cellulophaga lytica
CRH86348.1	Chlamydia trachomatis
KXK50582.1	Chloroflexi bacterium
OGO14047.1	Chloroflexi bacterium
OGO21664.1	Chloroflexi bacterium
OGO37781.1	Chloroflexi bacterium
OGO48922.1	Chloroflexi bacterium
EQI15153.1	Clostridioides difficile
AFS77817.1	Clostridium acidurici
ACL76030.1	Clostridium cellulolyticum
WP_15925145.1	Clostridium cellulolyticum
WP_19026902.1	Colwellia piezophila
AAZ26068.1	Colwellia psychrerythraea
AAZ28859.1	Colwellia psychrerythraea
WP_11042727.1	Colwellia psychrerythraea
ERM88230.1	Coprobacter fastidiosus
WP_22602726.1	Coprobacter fastidiosus
EHP42609.1	Cupriavidus basilensis
AFY29041.1	Cyanobium gracile
WP_15109489.1	Cyanobium gracile
EDY39186.1	Cyanobium sp.
WP_6911101.1	Cyanobium sp.
ACK69922.1	Cyanothece sp.
ACL44292.1	Cyanothece sp.
ADN16075.1	Cyanothece sp.
ADN17864.1	Cyanothece sp.
WP_12598867.1	Cyanothece sp.
WP_12627377.1	Cyanothece sp.
WP_13324141.1	Cyanothece sp.
WP_13334614.1	Cyanothece sp.
WP_19528756.1	Dasania marina
WP_19531305.1	Dasania marina
AFV06486.1	Dehalobacter sp.
PCJ78609.1	Dehalococcoidia bacterium
AGA70037.1	Desulfitobacterium dichloroeliminans
WP_15263008.1	Desulfitobacterium dichloroeliminans
ACL18657.1	Desulfitobacterium hafniense
WP_5808268.1	Desulfitobacterium hafniense
AFM39444.1	Desulfosporosinus acidiphilus
AFM39642.1	Desulfosporosinus acidiphilus

AFM41442.1	Desulfosporosinus acidiphilus
WP_14825457.1	Desulfosporosinus acidiphilus
WP_14825654.1	Desulfosporosinus acidiphilus
WP_14827440.1	Desulfosporosinus acidiphilus
EGW36218.1	Desulfosporosinus sp.
WP_9624553.1	Desulfosporosinus sp.
ACS80526.1	Desulfovibrio salexigens
AEE20941.1	Dokdonia sp.
WP_13752448.1	Dokdonia sp.
EGJ99214.1	Dysgonomonas gadei
WP_6798045.1	Dysgonomonas gadei
EHQ53638.1	Ectothiorhodospira sp.
WP_9306221.1	Eggerthella Actinobacteria
ACV56021.1	Eggerthella lenta
BAK44166.1	Eggerthella sp.
EGC90419.1	Eggerthella sp.
WP_13979416.1	Eggerthella sp.
WP_19015025.1	Elioraea tepidiphila
BAN69793.1	endosymbiont of
WP_43108529.1	endosymbiont of
OEF61668.1	Enterovibrio calviensis
OEE61532.1	Enterovibrio norvegicus
OEE90475.1	Enterovibrio norvegicus
SFQ05201.1	Enterovibrio norvegicus
WP_16959934.1	Enterovibrio norvegicus
WP_17016338.1	Enterovibrio Proteobacteria
ADN74271.1	Ferrimonas balearica
WP_13343577.1	Ferrimonas balearica
EEG43001.1	Flavobacteria bacterium
WP_8867824.1	Flavobacteria bacterium
EHO79959.1	Fusobacterium ulcerans
WP_5978753.1	Fusobacterium ulcerans
EES63658.2	Fusobacterium varium
EGH00070.1	gamma proteobacterium
WP_8172764.1	Gammaproteobacteria Gammaproteobacteria
WP_26852305.1	Geothrix fermentans
WP_26852554.1	Geothrix fermentans
CBL04394.1	Gordonibacter pamelaeae
WP_14966954.1	Gottschalkia acidurici
EOD77118.1	Grimontia indica
WP_2542577.1	Grimontia indica
WP_20408702.1	Hahella ganghwensis
ADQ13799.1	Halanaerobium hydrogeniformans
WP_13404905.1	Halanaerobium hydrogeniformans
EPC03278.1	Halomonas anticariensis
WP_16415392.1	Halomonas anticariensis
ERS86297.1	Halomonas sp.

WP_23006485.1	Halomonas sp.
EDQ32101.1	Hoeflea phototrophica
WP_7196449.1	Hoeflea phototrophica
WP_18426176.1	Hoeflea sp.
WP_5037910.1	Holophaga foetida
ABD54187.1	Jannaschia sp.
WP_11454394.1	Jannaschia sp.
WP_20593768.1	Kiloniella laminariae
WP_6592869.1	Kineosphaera limosa
BAJ33289.1	Kitasatospora setae
WP_14140580.1	Kitasatospora setae
KPV50590.1	Kouleothrix aurantiaca
EEE44608.1	Labrenzia alexandrii
WP_8191849.1	Labrenzia alexandrii
WP_20508030.1	Lamprocystis purpurea
WP_8554809.1	Leisingera daeponensis
WP_19296069.1	Leisingera Proteobacteria
KIC23138.1	Leisingera sp.
KIC49424.1	Leisingera sp.
KID09465.1	Leisingera sp.
ESA38583.1	Leptolyngbya sp.
WP_23070776.1	Leptolyngbya sp.
EQA44713.1	Leptospira broomii
WP_10571948.1	Leptospira broomii
EPG74209.1	Leptospira fainei
EQA38811.1	Leptospira inadai
OCW88351.1	Leptospira inadai
WP_20988026.1	Leptospira inadai
EQA55098.1	Leptospira kmetyi
WP_10573741.1	Leptospira kmetyi
EID99798.1	Leptospira licerasiae
WP_8592935.1	Leptospira licerasiae
EMJ98610.1	Leptospira sp.
WP_20770829.1	Leptospira sp.
EPG67275.1	Leptospira wolffii
WP_16544980.1	Leptospira wolffii
ACB32705.1	Leptothrix cholodnii
WP_12345467.1	Leptothrix cholodnii
WP_22952922.1	Leucothrix mucor
WP_20539233.1	Lewinella cohaerens
WP_75073805.1	Longilinea arvoryzae
ERT07515.1	Lyngbya aestuarii
WP_23066352.1	Lyngbya aestuarii
EAW38507.1	Lyngbya sp.
WP_9782974.1	Lyngbya sp.
EAW32933.1	marine gamma
WP_7224307.1	marine gamma

ADP99289.1	Marinobacter adhaerens
WP_14578680.1	Marinobacter adhaerens
EDM47252.1	Marinobacter algicola
WP_7154216.1	Marinobacter algicola
EHJ04812.1	Marinobacter manganoxydans
EHJ05040.1	Marinobacter manganoxydans
WP_8171991.1	Marinobacter manganoxydans
EMP54769.1	Marinobacter santoriniensis
WP_8939847.1	Marinobacter santoriniensis
WP_20681477.1	Marinobacterium rhizophilum
EAQ67255.1	Marinomonas sp.
WP_9832203.1	Marinomonas sp.
EAU54895.1	Mariprofundus ferrooxydans
EAU54908.1	Mariprofundus ferrooxydans
WP_9849409.1	Mariprofundus ferrooxydans
WP_18294348.1	Mariprofundus ferrooxydans
EAQ11294.1	Maritimibacter alkaliphilus
EAQ14647.1	Maritimibacter alkaliphilus
WP_8329168.1	Maritimibacter alkaliphilus
WP_8334411.1	Maritimibacter alkaliphilus
ADR22317.1	Marivirga tractuosa
WP_13454460.1	Marivirga tractuosa
WP_18467228.1	Meiothermus timidus
AGB43224.1	Mesorhizobium australicum
WP_15314696.1	Mesorhizobium australicum
ADV09911.1	Mesorhizobium ciceri
WP_13528606.1	Mesorhizobium ciceri
YP_4139961.1	Mesorhizobium ciceri
ANN55949.1	Mesorhizobium loti
BAB51264.1	Mesorhizobium loti
WP_10912606.1	Mesorhizobium loti
WP_19862339.1	Mesorhizobium loti
AEH85255.1	Mesorhizobium opportunistum
WP_13891996.1	Mesorhizobium opportunistum
WP_6326299.1	Mesorhizobium sp.
WP_16919118.1	Methylocystis parvus
WP_19866100.1	Methylovulum miyakonense
WP_10133555.1	Microbulbifer agarilyticus
WP_8478465.1	Nitrolancea hollandica
ADE15736.1	Nitrosococcus halophilus
WP_13033596.1	Nitrosococcus halophilus
ABA59400.1	Nitrosococcus oceani
WP_11331107.1	Nitrosococcus oceani
ADJ29698.1	Nitrosococcus watsonii
AEJ01607.1	Nitrosomonas sp.
EIZ78796.1	Novosphingobium sp.
WP_8995771.1	Novosphingobium sp.

EAT11057.1	Oceanobacter sp.
EAR60213.1	Oceanospirillum sp.
AGI71924.1	Octadecabacter arcticus
AGI73935.1	Octadecabacter arcticus
WP_15495061.1	Octadecabacter arcticus
WP_15496915.1	Octadecabacter arcticus
EHP45623.1	Odoribacter laneus
WP_9137739.1	Odoribacter laneus
ADY34018.1	Odoribacter splanchnicus
WP_13613209.1	Odoribacter splanchnicus
WP_7653120.1	Parabacteroides Bacteroidetes
EKN16551.1	Parabacteroides goldsteinii
AGT08312.1	Paracoccus aminophilus
AGH44204.1	Paraglaciecola psychrophila
WP_7643408.1	Paraglaciecola psychrophila
ELR65656.1	Photobacterium marinum
WP_7465580.1	Photobacterium marinum
CAG19776.1	Photobacterium profundum
EAS43655.1	Photobacterium profundum
WP_6231467.1	Photobacterium profundum
WP_11218100.1	Photobacterium profundum
BAM03112.1	Phycisphaera mikurensis
WP_12909180.1	Pirellula staleyi
WP_13110247.1	Planctopirus limnophila
EDM78911.1	Plesiocystis pacifica
WP_6971967.1	Plesiocystis pacifica
KKI99104.1	Prochlorothrix hollandica
WP_17712574.1	Prochlorothrix hollandica
BAN46568.1	Pseudomonas resinovorans
WP_16490770.1	Pseudomonas resinovorans
WP_17937445.1	Pseudomonas thermotolerans
ABM03505.1	Psychromonas ingrahamii
WP_11770065.1	Psychromonas ingrahamii
WP_8045841.1	Reinekea blandensis
EAR10115.1	Reinekea sp.
EDZ43148.1	Rhodobacteraceae bacterium
EEE37598.1	Rhodobacteraceae bacterium
WP_9829985.1	Rhodobacteraceae bacterium
WP_8563072.1	Rhodobacteraceae Proteobacteria
EAU53009.1	Rhodobacterales bacterium
EDZ46253.1	Rhodobacterales bacterium
PDH41214.1	Rhodothermaeota bacterium
PDH50325.1	Rhodothermaeota bacterium
AFY52941.1	Rivularia sp.
WP_15116519.1	Rivularia sp.
EBA14061.1	Roseobacter sp.
EDM72549.1	Roseobacter sp.

WP_7813115.1	Roseobacter sp.
EAQ25022.1	Roseovarius sp.
WP_13630312.1	Rubinisphaera brasiliensis
WP_10441831.1	Ruegeria conchae
AAV94248.1	Ruegeria pomeroyi
WP_11046692.1	Ruegeria pomeroyi
EEB72724.1	Ruegeria sp.
PCI18602.1	SAR202 cluster
WP_10584874.1	Schlesneria paludicola
ACZ07654.1	Sebaldella termitidis
WP_12860250.1	Sebaldella termitidis
EDQ02769.1	Shewanella benthica
ABZ77846.1	Shewanella halifaxensis
WP_12278367.1	Shewanella halifaxensis
ABO24850.1	Shewanella loihica
WP_11866781.1	Shewanella loihica
ABV88543.1	Shewanella pealeana
WP_12156444.1	Shewanella pealeana
ACJ27775.1	Shewanella piezotolerans
WP_20911153.1	Shewanella piezotolerans
WP_12325614.1	Shewanella Proteobacteria
ABV38156.1	Shewanella sediminis
WP_12143886.1	Shewanella sediminis
BAJ03438.1	Shewanella violacea
WP_13052733.1	Shewanella violacea
ACA87278.1	Shewanella woodyi
ADE10318.1	Sideroxydans lithotrophicus
WP_13028217.1	Sideroxydans lithotrophicus
ACZ40634.1	Sphaerobacter thermophilus
WP_41400480.1	Sphaerobacter thermophilus
ADN02815.1	Spirochaeta thermophila
AEJ62183.1	Spirochaeta thermophila
WP_13314654.1	Spirochaeta thermophila
WP_14625506.1	Spirochaeta thermophila
EDY47343.1	Streptomyces clavuligerus
EFG05001.1	Streptomyces clavuligerus
WP_3952745.1	Streptomyces clavuligerus
WP_20553940.1	Streptomyces scabrisporus
ACZ87948.1	Streptosporangium roseum
BAN35971.1	Sulfuricella denitrificans
WP_9205168.1	Sulfuricella denitrificans
ADR35090.1	Sulfuricurvum kujiense
WP_13461287.1	Sulfuricurvum kujiense
ACN98151.1	Sulfurihydrogenibium azorense
WP_12673477.1	Sulfurihydrogenibium azorense
ACD66493.1	Sulfurihydrogenibium sp.
WP_12459567.1	Sulfurihydrogenibium sp.

EDZ61136.1	Sulfurimonas gotlandica
EHP30902.1	Sulfurimonas gotlandica
WP_8340197.1	Sulfurimonas gotlandica
ADY56004.1	Syntrophobotulus glycolicus
WP_13624872.1	Syntrophobotulus glycolicus
EHL88295.1	Tannerella sp.
WP_9317002.1	Tannerella sp.
CCU71633.1	Thalassolituus oleivorans
WP_15486370.1	Thalassolituus oleivorans
ENO97417.1	Thauera phenylacetica
WP_4361181.1	Thauera phenylacetica
WP_4307711.1	Thauera Proteobacteria
ACR02493.1	Thauera sp.
WP_83461725.1	Thermanaerothrix daxensis
WP_38047055.1	Thermoanaerobaculum aquaticum
SNB65073.1	Thermoflexus hugenholtzii
WP_88571165.1	Thermoflexus hugenholtzii
WP_38057787.1	Thermus amyloliquefaciens
WP_71676671.1	Thermus brockianus
WP_38047563.1	Thermus caliditerrae
WP_15717826.1	Thermus scotoductus
WP_38030997.1	Thermus sp
WP_39458450.1	Thermus sp
WP_38041695.1	Thermus tengchongensis
WP_14510533.1	Thermus thermophilus
AGA33196.1	Thioalkalivibrio nitratireducens
WP_15258330.1	Thioalkalivibrio nitratireducens
ADC72022.1	Thioalkalivibrio sp.
WP_12982907.1	Thioalkalivibrio sp.
WP_17926265.1	Thioalkalivibrio sp.
WP_18141004.1	Thioalkalivibrio sp.
WP_18145552.1	Thioalkalivibrio sp.
WP_18169886.1	Thioalkalivibrio sp.
WP_18176711.1	Thioalkalivibrio sp.
WP_18862286.1	Thioalkalivibrio sp.
WP_18864527.1	Thioalkalivibrio sp.
WP_18870747.1	Thioalkalivibrio sp.
WP_18872982.1	Thioalkalivibrio sp.
WP_18875158.1	Thioalkalivibrio sp.
WP_18875609.1	Thioalkalivibrio sp.
WP_18877481.1	Thioalkalivibrio sp.
WP_18880320.1	Thioalkalivibrio sp.
WP_18934977.1	Thioalkalivibrio sp.
WP_18941052.1	Thioalkalivibrio sp.
WP_18947929.1	Thioalkalivibrio sp.
WP_18949363.1	Thioalkalivibrio sp.
WP_19022810.1	Thioalkalivibrio sp.

WP_19558102.1	Thioalkalivibrio sp.
WP_19562583.1	Thioalkalivibrio sp.
WP_19568301.1	Thioalkalivibrio sp.
WP_19570642.1	Thioalkalivibrio sp.
WP_19571351.1	Thioalkalivibrio sp.
WP_19583880.1	Thioalkalivibrio sp.
WP_19591361.1	Thioalkalivibrio sp.
WP_19593258.1	Thioalkalivibrio sp.
WP_19611829.1	Thioalkalivibrio sp.
WP_19627000.1	Thioalkalivibrio sp.
WP_19628124.1	Thioalkalivibrio sp.
WP_19642372.1	Thioalkalivibrio sp.
WP_20146808.1	Thioalkalivibrio sp.
ACL71586.1	Thioalkalivibrio sulfidiphilus
ACL72045.1	Thioalkalivibrio sulfidiphilus
WP_12637074.1	Thioalkalivibrio sulfidiphilus
WP_12637529.1	Thioalkalivibrio sulfidiphilus
WP_18232636.1	Thioalkalivibrio thiocyanodenitrificans
WP_18234051.1	Thioalkalivibrio thiocyanodenitrificans
OOC48798.1	Thioalkalivibrio versutus
WP_18650065.1	Thioalkalivibrio versutus
AAZ96815.1	Thiobacillus denitrificans
WP_11311374.1	Thiobacillus denitrificans
WP_18078921.1	Thiobacillus denitrificans
WP_18507680.1	Thiobacillus thioparus
EGV16401.1	Thiocapsa marina
WP_7195218.1	Thiocapsa marina
AFL72274.1	Thiocystis violascens
WP_14776782.1	Thiocystis violascens
AGA89667.1	Thioflavicoccus mobilis
WP_15279813.1	Thioflavicoccus mobilis
EGV33725.1	Thiorhodococcus drewsii
WP_7039130.1	Thiorhodococcus drewsii
EIJ32887.1	Thiothrix nivea
WP_2706850.1	Thiothrix nivea
AFM12566.1	Turneriella parva
WP_14803075.1	Turneriella parva
BAL55967.1	uncultured planctomycete
EDN57141.1	Vibrio antiquarius
WP_6742301.1	Vibrio antiquarius
EGA66677.1	Vibrio brasiliensis
WP_6878503.1	Vibrio brasiliensis
OED96668.1	Vibrio breoganii
OEF82948.1	Vibrio breoganii
WP_17031818.1	Vibrio breoganii
OEF02639.1	Vibrio crassostreae
WP_17065248.1	Vibrio crassostreae

OEF33005.1	Vibrio cyclitrophicus
WP_16785709.1	Vibrio cyclitrophicus
EEZ86426.1	Vibrio harveyi
WP_17190843.1	Vibrio harveyi
OEF14099.1	Vibrio kanaloae
WP_17056989.1	Vibrio kanaloae
EEX94887.1	Vibrio orientalis
EGU52981.1	Vibrio orientalis
WP_4412950.1	Vibrio orientalis
AGQ93411.1	Vibrio parahaemolyticus
WP_17447629.1	Vibrio parahaemolyticus
WP_20842042.1	Vibrio parahaemolyticus
WP_10452121.1	Vibrio rotiferianus
EDL55345.1	Vibrio shilonii
WP_6069405.1	Vibrio shilonii
AEX24264.1	Vibrio sp.
WP_14234108.1	Vibrio sp.
WP_17635336.1	Vibrio sp.
EAP95374.1	Vibrio splendidus
WP_4736389.1	Vibrio splendidus
WP_17092560.1	Vibrio splendidus
CAV19313.1	Vibrio tasmaniensis
OEF54308.1	Vibrio tasmaniensis
WP_12604459.1	Vibrio tasmaniensis
WP_17104152.1	Vibrio tasmaniensis
WP_19961242.1	Woodsholea maritima
ETA79758.1	Youngiibacter fragilis

Supplementary Table S2: List of archaeal CISD sequences

Archaeal CDGSH sequences	
**Accession no.**	**Organism Name**
WP_013776315.1	Acidianus hospitalis
WP_013266756.1	Acidilobus saccharovorans
WP_048102028.1	Acidiplasma
WP_022541719.1	Aeropyrum camini
WP_048816623.1	Caldisphaera lagunensis
WP_012186123.1	Caldivirga maquilingensis
WP_066793800.1	Caldivirga sp MU80
WP_023395167.1	Candidatus Halobonum tyrrellensis
WP_048111451.1	Candidatus Methanoplasma termitum
WP_007551175.1	Candidatus Nitrosoarchaeum koreensis
WP_010189805.1	Candidatus Nitrosoarchaeum limnia
WP_048117497.1	Candidatus Nitrosopumilus adriaticus
WP_021789221.1	Cuniculiplasma divulgatum
WP_009887068.1	Ferroplasma
WP_049972192.1	Haladaptatus cibarius
WP_076431795.1	Haladaptatus litoreus
WP_076433289.1	Haladaptatus litoreus
WP_007978056.1	Haladaptatus paucihalophilus
WP_066139249.1	Haladaptatus sp R4
WP_069451248.1	Haladaptatus sp W1
WP_008417031.1	Halalkalicoccus jeotgali
WP_066382532.1	Halalkalicoccus paucihalophilus
WP_050049010.1	Halanaeroarchaeum sulfurireducens
WP_020222023.1	Halarchaeum acidiphilum
WP_073307495.1	Halobaculum gomorrense
WP_007141007.1	Halobiforma lacisalsi
WP_049920262.1	Halobiforma nitratireducens
WP_049996532.1	Halococcus sediminicola
WP_071932602.1	Halodesulfurarchaeum formicicum
WP_070364270.1	Halodesulfurarchaeum formicicum
WP_006053467.1	Halogeometricum borinquense
WP_074874472.1	Halolamina pelagica
WP_054584337.1	Halolamina pelagica
WP_049981374.1	Halolamina rubra
WP_053948499.1	Halolamina sediminis
WP_079233080.1	Halolamina sp CBA1230
WP_018257272.1	Halomicrobium katesii
WP_015762517.1	Halomicrobium mukohataei
WP_014051935.1	halophilic archaeon DL31
WP_049922601.1	Halopiger djelfimassiliensis
WP_049990126.1	Halopiger salifodinae
WP_013879458.1	Halopiger xanaduensis
WP_049935503.1	Haloplanus natans
WP_011571670.1	Haloquadratum walsbyi

WP_077204814.1	Halorientalis sp IM1011
WP_004595828.1	Halorubrum
WP_007993349.1	Halorubrum arcis
WP_008442536.1	Halorubrum californiense
WP_049929591.1	Halorubrum ezzemoulense
WP_050033186.1	Halorubrum halophilum
WP_049908553.1	Halorubrum hochstenium
WP_049912263.1	Halorubrum kocurii
WP_015910050.1	Halorubrum lacusprofundi
WP_008003440.1	Halorubrum lipolyticum
WP_050024124.1	Halorubrum saccharovorum
WP_004045862.1	Halorubrum saccharovorum
WP_048078281.1	Halorubrum sp AJ67
WP_049982221.1	Halorubrum sp BV1
WP_066414509.1	Halorubrum sp SAH-A6
WP_086219209.1	Halorubrum sp SD612
WP_086221068.1	Halorubrum sp SD612
WP_086215803.1	Halorubrum sp SD683
WP_017343226.1	Halorubrum sp T3
WP_049906233.1	Halorubrum tebenquichense
WP_053771220.1	Halorubrum tropicale
WP_049951710.1	Halostagnicola larsenii
WP_050050167.1	Halostagnicola sp A56
WP_049965073.1	Haloterrigena jeotgali
WP_008010701.1	Haloterrigena limicola
WP_049911531.1	Haloterrigena limicola
WP_066303711.1	Haloterrigena mahii
WP_076147084.1	Haloterrigena saccharevitans
WP_008892751.1	Haloterrigena salina
WP_006650002.1	Haloterrigena thermotolerans
WP_012941689.1	Haloterrigena turkmenica
WP_048079476.1	Marine Group I
WP_048071020.1	Marine Group I thaumarchaeote SCGC AAA799-P11
WP_011921411.1	Metallosphaera sedula
WP_009072948.1	Metallosphaera yellowstonensis
WP_048080982.1	Methanobacterium
WP_071907851.1	Methanobacterium congolense
WP_004032061.1	Methanobacterium formicicum
WP_048072312.1	Methanobacterium formicicum
WP_013643737.1	Methanobacterium lacus
WP_013826864.1	Methanobacterium paludis
WP_013825376.1	Methanobacterium paludis
WP_069581839.1	Methanobacterium sp A39
WP_023992635.1	Methanobacterium sp MB1
WP_048192762.1	Methanobacterium sp SMA-27
WP_054835936.1	Methanobrevibacter arboriphilus
WP_080459248.1	Methanobrevibacter arboriphilus

WP_067089782.1	Methanobrevibacter curvatus
WP_067258381.1	Methanobrevibacter cuticularis
WP_066973840.1	Methanobrevibacter filiformis
WP_083495826.1	Methanobrevibacter millerae
WP_081477291.1	Methanocella arvoryzae
WP_011500268.1	Methanococcoides burtonii
WP_011498660.1	Methanococcoides burtonii
WP_048193743.1	Methanococcoides methylutens
WP_048204675.1	Methanococcoides methylutens
WP_048194737.1	Methanococcoides methylutens
WP_048205523.1	Methanococcoides methylutens
WP_011971960.1	Methanococcus vannielii
WP_074370700.1	Methanoculleus chikugoensis
WP_048180103.1	Methanoculleus sediminis
WP_083475320.1	Methanogenium cariaci
WP_072560411.1	Methanohalophilus halophilus
WP_013036951.1	Methanohalophilus mahii
WP_072359189.1	Methanohalophilus portucalensis
WP_023846582.1	Methanolobus tindarius
WP_023846254.1	Methanolobus tindarius
WP_019177401.1	Methanomassiliicoccus luminyensis
WP_081579869.1	Methanomassiliicoccus luminyensis
WP_015324563.1	Methanomethylovorans hollandica
WP_012107888.1	Methanoregula boonei
WP_013718431.1	Methanosaeta concilii
WP_014586911.1	Methanosaeta harundinacea
WP_048155293.1	Methanosarcina
WP_048128957.1	Methanosarcina
WP_048140578.1	Methanosarcina
WP_011021023.1	Methanosarcina acetivorans
WP_011021684.1	Methanosarcina acetivorans
WP_011307268.1	Methanosarcina barkeri
WP_048118106.1	Methanosarcina barkeri
WP_048107164.1	Methanosarcina barkeri
WP_048103491.1	Methanosarcina barkeri
WP_011308483.1	Methanosarcina barkeri
WP_080943665.1	Methanosarcina barkeri
WP_054297746.1	Methanosarcina flavescens
WP_048139517.1	Methanosarcina horonobensis
WP_048126757.1	Methanosarcina lacustris
WP_048126054.1	Methanosarcina lacustris
WP_048045253.1	Methanosarcina mazei
WP_048040775.1	Methanosarcina mazei
WP_048037902.1	Methanosarcina mazei
WP_011034932.1	Methanosarcina mazei
WP_048039071.1	Methanosarcina mazei
WP_048046450.1	Methanosarcina mazei

WP_048048029.1	Methanosarcina mazei
WP_048041442.1	Methanosarcina mazei
WP_048170480.1	Methanosarcina siciliae
WP_048051731.1	Methanosarcina soligelidi
WP_048160643.1	Methanosarcina sp 1HA22
WP_048135064.1	Methanosarcina sp 1HT1A1
WP_082106041.1	Methanosarcina sp 1HT1A1
WP_048172530.1	Methanosarcina sp 2HA1B4
WP_048157694.1	Methanosarcina sp Kolksee
WP_048158242.1	Methanosarcina sp Kolksee
WP_048178702.1	Methanosarcina sp MTP4
WP_082090559.1	Methanosarcina sp MTP4
WP_048166474.1	Methanosarcina thermophila
WP_048122560.1	Methanosarcina vacuolata
WP_048123830.1	Methanosarcina vacuolata
WP_012617838.1	Methanosphaerula palustris
WP_012617934.1	Methanosphaerula palustris
WP_012618116.1	Methanosphaerula palustris
WP_012619089.1	Methanosphaerula palustris
WP_048901179.1	Methanothermobacter marburgensis
WP_048176473.1	Methanothermobacter sp CaT2
WP_048060766.1	Methanothermobacter thermautotrophicus
WP_074358693.1	Methanothermobacter wolfeii
WP_006166289.1	Natrialba chahannaoensis
WP_086887728.1	Natrialbaceae archaeon JW/NM-HA 15
WP_008456977.1	Natrinema
WP_007107636.1	Natrinema altunense
WP_006187219.1	Natrinema pallidum
WP_006182629.1	Natrinema pellirubrum
WP_006429052.1	Natrinema versiforme
WP_005580564.1	Natronobacterium gregoryi
WP_005557478.1	Natronococcus amylolyticus
WP_005554376.1	Natronococcus amylolyticus
WP_049893246.1	Natronococcus jeotgali
WP_015319633.1	Natronococcus occultus
WP_054863213.1	Natronolimnobius baerhuensis
WP_007259150.1	Natronolimnobius innermongolicus
WP_006065050.1	Natronorubrum bangense
WP_008160831.1	Natronorubrum sulfidifaciens
WP_076609781.1	Natronorubrum thiooxidans
WP_006087814.1	Natronorubrum tibetense
WP_014963490.1	Nitrosopumilus
WP_014964456.1	Nitrosopumilus
WP_075054790.1	Nitrososphaera viennensis
WP_048147879.1	Palaeococcus ferrophilus
WP_048059454.1	Picrophilus
WP_058371553.1	Pyrodictium occultum

WP_054839292.1	Sulfolobus metallicus
WP_054846334.1	Sulfolobus sp JCM 16833
WP_048196951.1	Thaumarchaeota archaeon N4
WP_010900511.1	Thermoplasma acidophilum
WP_010916426.1	Thermoplasma volcanium
WP_069807158.1	Vulcanisaeta thermophila

Supplementary Table S3: BEAST model test table.

Molecular Clock model, Population Growth model	Posterior		Likelihood		Rank
	Mean	ESS	Mean	ESS	
UCLN, Constant	-7174.65	297	-4207.746	1099	1
UCLN, Exponential	-7162.1	312	-4208.649	570	2
UCLN, BSKY (Rejected due to low ESS)	-9564.1	58	-4196.684	408	3
Strict, BSKY (Rejected due to low ESS)	-8037.16	88	-4236.615	398	4
Strict, Constant (Rejected due to low ESS)	-5673.29	143	-4301.066	566	5
Strict, Exponential (Rejected due to higher posterior and likelihood values)	-5648.28	279	-4306.91	1047	6

UCLN = uncorrelated log-normal clock
BSKY = Bayesian skyline
ESS = Estimated sample size
